# Cell-specific network constructed by single-cell RNA sequencing data

**DOI:** 10.1093/nar/gkz172

**Published:** 2019-03-13

**Authors:** Hao Dai, Lin Li, Tao Zeng, Luonan Chen

**Affiliations:** 1 Center for Excellence in Molecular Cell Science, Institute of Biochemistry and Cell Biology, Chinese Academy of Sciences, Shanghai 200031, China; 2 Center for Excellence in Animal Evolution and Genetics, Chinese Academy of Sciences, Kunming 650223, China; 3 School of Life Science and Technology, ShanghaiTech University, Shanghai 201210, China; 4 Shanghai Research Center for Brain Science and Brain-Inspired Intelligence, Shanghai 201210, China

## Abstract

Single-cell RNA sequencing (scRNA-seq) is able to give an insight into the gene–gene associations or transcriptional networks among cell populations based on the sequencing of a large number of cells. However, traditional network methods are limited to the grouped cells instead of each single cell, and thus the heterogeneity of single cells will be erased. We present a new method to construct a cell-specific network (CSN) for each single cell from scRNA-seq data (i.e. one network for one cell), which transforms the data from ‘unstable’ gene expression form to ‘stable’ gene association form on a single-cell basis. In particular, it is for the first time that we can identify the gene associations/network at a single-cell resolution level. By CSN method, scRNA-seq data can be analyzed for clustering and pseudo-trajectory from network perspective by any existing method, which opens a new way to scRNA-seq data analyses. In addition, CSN is able to find differential gene associations for each single cell, and even ‘dark’ genes that play important roles at the network level but are generally ignored by traditional differential gene expression analyses. In addition, CSN can be applied to construct individual network of each sample bulk RNA-seq data. Experiments on various scRNA-seq datasets validated the effectiveness of CSN in terms of accuracy and robustness.

## INTRODUCTION

Single-cell RNA sequencing (scRNA-seq) provides a high-throughput method to measure and compare the levels of gene expression at single cell resolution (1,2). The heterogeneity and functional diversity among cell populations can be revealed and new cell types with distinct functions may be discovered (3–5). Recent studies provide many accurate and robust computational methods to identify new cell types by solving the problems of outlier cell populations, transcript amplification noise and dropout events in scRNA-seq (6–9). However, most of these methods mainly focused on the analyses of gene expression levels, while scRNA-seq may give more information of an insight into the gene–gene associations or transcriptional networks based on the sequencing of hundreds to thousands of cells. Many biological processes such as co-expression, transcriptional regulation, DNA modification, function of non-coding RNA involve the problems of gene–gene associations, whose understanding and explanation will greatly help to reveal the mystery of life.

The biological system in a cell is generally a nonlinear dynamical system. From dynamical viewpoint, gene expressions are variables of such a system and may be different if measured at different time points or conditions even for the same cell. In contrast, it is gene associations or transcriptional networks that result in the measured gene expression patterns, and thus is a stable form against the time and condition. Therefore, the network of a cell can more reliably characterize the biological system or state of the cell. Traditional network methods (10,11) are useful to analyze the gene–gene associations from scRNA-seq data, but the cells should be clustered or classified in advance, and the network is usually limited to be constructed for the grouped cells instead of each single cell. Thus as a result, the heterogeneity of single cells will be erased. In addition, nonlinear associations among genes are usually hard to be identified, in particular for single cell.

In this study, we propose a new computational method to construct a cell-specific network (CSN) on a single-cell basis from scRNA-seq data, which means one network for one cell. The input data of CSN method is just the original gene expression matrix (GEM) of all cells, and the output is a series of CSNs in which nodes are genes and edges are gene–gene associations. CSN method is derived from our new theoretical model based on statistical dependency, which can be viewed as data transformation from the ‘unstable’ gene expression data to the ‘stable’ gene association data. Computationally, we do not need to cluster or classify the cells at first, and theoretically both linear and nonlinear associations among genes can be identified. By CSN method, it is for the first time that we can identify the gene–gene associations or transcriptional networks at a single-cell level. To facilitate the analysis, a network degree matrix (NDM) is further constructed from CSNs, in which each element is not the gene expression level, but the number of edges connected to each gene in each CSN. NDM embodies the network features and reflects the importance of each gene in the network, which has the same number of rows and columns as the original GEM, so that it can be analyzed for cell clustering and pseudo-trajectory construction by any existing scRNA-seq method, which opens a new way to analyze scRNA-seq data from network perspective. Experiments on various scRNA-seq datasets illustrated that NDM had better performances than original GEM among most clustering and pseudo-trajectory methods in terms of accuracy and robustness. In addition, CSN is able to find key genes or even ‘dark’ genes that have significant difference between case and control samples not in a gene expression level but in a network degree level. Generally, our CSN method provides a new way to analyze the scRNA-seq data, and in particular extracts richer information of biological systems at the network level. Moreover, CSN can be directly applied to construct individual network of each single sample from bulk RNA-seq data.

## MATERIALS AND METHODS

### Construction of cell-specific network

**Figure 1.**
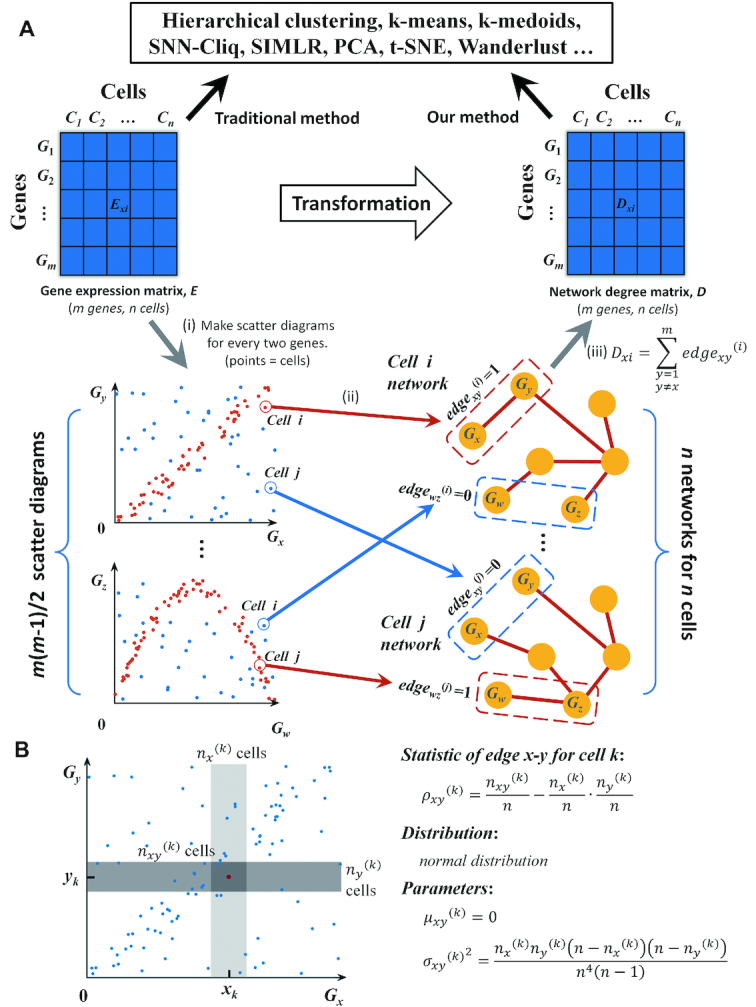
Schematic illustration of CSN and NDM construction and our statistic model. (**A**) CSN and NDM construction. (i) Make scatter diagrams for every two genes, where each point represents a cell, and *x*- and *y*-values are the expression values of the two genes in the *n* cells. Then *m* genes lead to *m* (*m* – 1)/2 scatter diagrams. (ii) In the scatter diagram of genes *x* and *y*, the plot *i* with red color means there is an edge between genes *x* and *y* in the cell *i* network based on our statistic model, and if the plot is blue, there is no edge. Then, we can construct *n* cell-specific networks corresponding to *n* cells, respectively. (iii) By counting the number of edges connected to each gene in each CSN, we can get the network degree matrix, which is still comprised of *m* rows and *n* columns, as the same as GEM, and thus it can be analyzed by any existing method. (**B**) Our statistic model for edge between genes *x* and *y*. Near the plot or cell *k*, make the light and medium grey boxes to represent the neighborhood of *x_k_* and *y_k_* respectively. The intersection of two boxes is the dark grey box, which represents the neighborhood of (*x_k_, y_k_*). The number of plots in the light, medium and dark grey boxes is *n_x_*^(^*^k^*^)^, *n_y_*^(^*^k^*^)^ and *n_xy_*^(^*^k^*^)^ respectively. Design the statistic as *ρ_xy_*^(^*^k^*^)^. If *x* and *y* are independent of each other, the statistic follows normal distribution and the mean value and variance can be calculated. If the statistic *ρ_xy_*^(^*^k^*^)^ is larger than a significant level, label plot *k* with red color, which means there is an edge between *x* and *y* in cell *k*; otherwise there is no edge.

In this paper, we propose a new method with a statistical model which constructs a cell-specific network (CSN) for each single cell from a scRNA-seq data. If the dataset comprises of *m* genes and *n* cells, we will construct *n* CSNs corresponding to the *n* cells, and in each CSN, there are *m* nodes corresponding to the *m* genes and the edges are gene–gene associations without direction (Figure 1A). The value of each edge is 1 or 0, which represents if or not two genes interact with each other. In this work, we assume that each single cell is characterized by its gene association network. In other words, due to the difference of cell types, two genes may have association in some cells but not in the other cells.

The gene–gene association is determined by the statistical independency of two genes. In probability theory, if two variables are independent of each other, the joint density function is equal to the product of two marginal density functions, which means

(1) \begin{equation*}f(x,y) = {f_X}(x) \cdot {f_Y}(y)\end{equation*}

where *f_X_* (*x*) and *f_Y_* (*y*) are marginal density functions of *x* and *y* respectively, and *f* (*x, y*) is joint density function.

Equation (1) is a global measurement of statistical independency, which is a necessary and sufficient condition. In this paper, we derive a local measurement from Equation (1) that is defined as *f*(*x_k_, y_k_*) – *f_X_*(*x_k_*) *f_Y_*(*y_k_*), which measures the independency of genes *x* and *y* in cell *k*. To estimate the values of *f_X_*(*x_k_*), *f_Y_*(*y_k_*) and *f*(*x_k_, y_k_*), we make a scatter diagram based on the expression values of genes *x* and *y*, in which each plot represents a cell, and then we draw three boxes near the plot *k* to represent the neighborhood of *x_k_, y_k_* and (*x_k_, y_k_*) respectively (Figure 1B), in which the number of plots are *n_x_*^(^*^k^*^)^, *n_y_*^(^*^k^*^)^ and *n_xy_*^(^*^k^*^)^. Then, we can substitute the probability by the frequency numerically.

(2) \begin{eqnarray*}&&{f_X}({x_k}) \approx \frac{{{n_x}^{(k)}}}{n},\,\,\,\,{f_Y}({y_k}) \approx \frac{{{n_y}^{(k)}}}{n},\nonumber\\ && \,\,\,\,\,\,\,\,\,\,\,\,\,\,\,\,\,\,\,\,\, f({x_k},{y_k}) \approx \frac{{{n_{xy}}^{(k)}}}{n}\end{eqnarray*}

where *n* is the total number of plots/cells.

Then, we design a statistic for genes *x, y* of cell *k* as

(3) \begin{equation*}{\rho _{xy}}^{(k)} = \frac{{{n_{xy}}^{(k)}}}{n} - \frac{{{n_x}^{(k)}}}{n} \cdot \frac{{{n_y}^{(k)}}}{n}\end{equation*}

*n_x_*^(^*^k^*^)^ and *n_y_*^(^*^k^*^)^ are predetermined integers (< *n*), and thus the statistic *ρ_xy_*^(^*^k^*^)^ is only changed with *n_xy_*^(^*^k^*^)^. In particular, we set *n_x_*^(^*^k^*^)^ = *n_y_*^(^*^k^*^)^ = 0.1*n* in this work, and *n_x_*^(^*^k^*^)^ and *n_y_*^(^*^k^*^)^ are both proportional to the sample size *n*. In other words, we first draw the two boxes near *x_k_* and *y_k_* based on the predetermined *n_x_*^(^*^k^*^)^ and *n_y_*^(^*^k^*^)^, and then we can straightforwardly have the third box, which is simply the intersection of the previous two boxes (Figure 1B). Thus, we can obtain the value of *n_xy_*^(^*^k^*^)^ by counting the plots in the third box, thereby testing the criterion of Equation (3).

**Figure 2.**
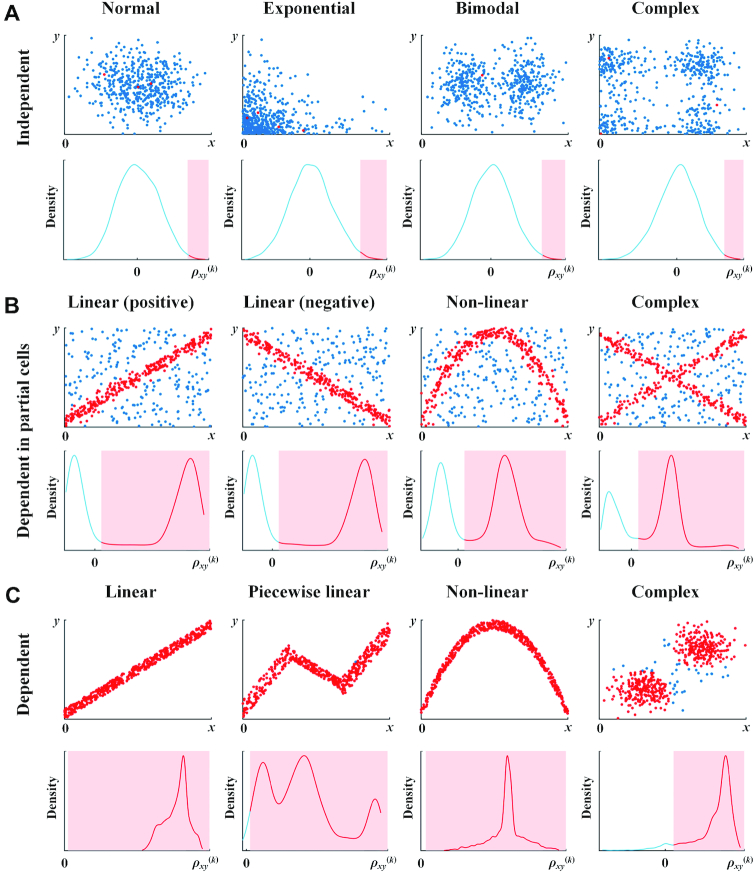
Probability density functions of the statistic *ρ_xy_*^(^*^k^*^)^ when (**A**) genes *x* and *y* are independent of each other; (**B**) genes *x* and *y* are dependent in partial cells; (**C**) genes *x* and *y* are dependent in all cells. Red plots in the scatter diagrams of genes *x* and *y* represent that the statistic *ρ_xy_*^(^*^k^*^)^ in these plots is larger than the significant level of 0.01 and *edge_xy_*^(^*^k^*^)^ = 1, which corresponds to the red area in the density function graphs. Blue plots represent that *ρ_xy_*^(^*^k^*^)^ in these plots is smaller than the significant level of 0.01 and *edge_xy_*^(^*^k^*^)^ = 0, which corresponds to the blue lines in the density function graphs. Sample size *n* = 500, and *n_x_*^(^*^k^*^)^ = *n_y_*^(^*^k^*^)^ = 0.1*n*.

The range of the statistic is -1 to 1, and it can be proved (Supplementary Note S1) that if *x* and *y* are independent of each other, the statistic *ρ_xy_*^(^*^k^*^)^ approximately follows normal distribution (Figure 2A) and the mean value and standard deviation are

(4) \begin{eqnarray*}{\mu _{xy}}^{(k)} &=& 0, \nonumber\\ {\sigma _{xy}}^{(k)} &=& \sqrt{\frac{{{{n_x}^{(k)}{n_y}^{(k)}(n - {n_x}^{(k)})(n - {n_y}^{(k)})} }}{n^4{(n-1)}}} \end{eqnarray*}

We normalized *ρ_xy_*^(^*^k^*^)^ as

(5) \begin{eqnarray*}{\hat{\rho }_{xy}}^{\,\,\,\,\,\,\,(k)} &=& \frac{{{\rho _{xy}}^{(k)} - {\mu _{xy}}^{(k)}}}{{{\sigma _{xy}}^{(k)}}}\nonumber\\ &=& \frac{{\sqrt {n - 1} \cdot (n \cdot {n_{xy}}^{(k)} - {n_x}^{(k)}{n_y}^{(k)})}}{{\sqrt {{n_x}^{(k)}{n_y}^{(k)}(n - {n_x}^{(k)})(n - {n_y}^{(k)})} }}\end{eqnarray*}

If genes *x* and *y* are independent of each other, this normalized statistic follows standard normal distribution and the mean value and variance for the *n* cells are 0 and 1 respectively.

We conducted the numerical simulation on the statistic *ρ_xy_*^(^*^k^*^)^ for various dependent relations. As shown in Figure 2, clearly if genes *x* and *y* are independent of each other, no matter which distributions the genes follow, the statistic *ρ_xy_*^(^*^k^*^)^ approximates to normal distribution (Figure 2A). On the other hand, if *x* and *y* are dependent in partial cells and independent in the other cells, no matter if the dependency relation is positive or negative, linear or nonlinear, simple or complex, the statistic shows double crest in the density function, and has larger values than the significant level in the cells with the dependent gene pairs (red plots in Figure 2B), and has smaller values in the cells with the independent gene pairs (blue plots in Figure 2B). If genes *x* and *y* are dependent in all cells, no matter which dependency relation is, the statistic of most cells is much larger than the significant level (Figure 2C). As a summary, the statistic *ρ_xy_*^(^*^k^*^)^ is a good measurement to distinguish the cells with dependent gene pairs and independent gene pairs in a reliable manner. In other words, we can analyze the gene associations at a single-cell level just by this statistic. The detailed descriptions for the statistic from both theoretical and computational viewpoints are also given in Supplementary Note S1.

Thus, to construct a network, by using the statistic with our statistical model Equation (5), we take the following hypothesis test (one-side test) for the genes association (edge *x*–*y* in the network of cell *k*):

*H*_0_ (null hypothesis): genes *x* and *y* are independent in cell *k*.

*H*_1_ (alternative hypothesis): genes *x* and *y* are associated with each other in cell *k*.

If the normalized statistic of Equation (5) is larger than a significant level, we will reject the null hypothesis and *edge_xy_*^(^*^k^*^)^ = 1, otherwise *edge_xy_*^(^*^k^*^)^ = 0. In this work, the significant level is set as 0.01. After repeating this process for all gene pairs and all cells, we can get *n* CSNs for *n* cells at last. As CSNs are only constructed from the gene expression matrix and we do not need to classify or cluster the cells at first. Thus, this method is an unsupervised network construction method.

Note that if point/cell *k* is an outlier in the scatter diagram of genes *x* and *y, edge_xy_*^(^*^k^*^)^ is equal to 0 because of the small value of *n_xy_*^(^*^k^*^)^, which means it is hard to find edges for outlier samples/cells.

It should be also noted that the zero expression of a gene is meaningful from a network viewpoint because the zero expression may come from the inhibition or negative regulation of another gene. Thus, even if the expression of gene *x* in cell *k* is zero, we may still find an edge between *x* and another gene. However, in scRNA-seq data, most zeros may result from the experimental problems, which are meaningless in biology and may produce errors in the data analysis. Hence, in this work, we treat the zeros in the following way: (1) If we cannot distinguish whether or not the zeros result from the zero-expression or the experimental problems, we just use all genes to construct CSNs, specifically, *edge_xy_*^(^*^k^*^)^ is set to 0 when *x_k_* = 0 or *y_k_* = 0 without the consideration of the statistic. (2) If we know that the zeros result from the zero-expression, *edge_xy_*^(^*^k^*^)^ is determined by the statistic.

### Network analysis of CSN

CSN provides a method to analyze the gene–gene associations at single-cell level. Based on the normalized statistic of two genes, differential analyses of gene–gene associations can be performed among different cell types by statistical test, and then we may find the two genes are associated/interacted in some cell types and independent in the other cell types. In other words, we may find the marker edges in some cell types. These edges may come from gene regulation, co-expression, alternative splicing and so on. Though our method cannot provide more details of the gene–gene associations, it still provides the biologists many important clues for further research.

CSN also provides a method to find the key genes from network perspective. As the key regulatory genes usually influence the expression of many other genes, there will be more edges connected to the key regulatory genes in CSNs, and thus the network degree of these genes will be higher. By calculating the number of edges connected to each gene (i.e. network degree) in each CSN, we can select the genes with the highest degrees in each cell or each cell type, which represent the key genes from network perspective and instruct the biologists in gene regulation studies.

### Network degree matrix from CSN

CSNs can be used for various biological studies at the network level, but the number of features describing a network for most scRNA-seq analyses is quite large. If there are *m* genes, there will be m*(*m*-1)/2 gene pairs or features. In this paper, we further transfer CSNs to a network degree matrix (NDM) to embody the network features and reduce the dimensions simultaneously although we can directly use the CSNs (or reduce the CSNs in other way) for clustering analysis. For gene *x* in the network of cell *k*

(6) \begin{equation*}{{\bf ND}}{{{\bf M}}_{xk}} = \sum\limits_{y = 1,y \ne x}^m {edg{e_{xy}}^{(k)}} \end{equation*}

Then we can get a matrix NDM with *m* × *n* elements. In this work, we will further normalize the NDM to make each cell has the same number of network degrees, which is shown in Equation (S-4) of Supplementary Note S2. The normalization is able to improve the robustness and helps to the comparison of the cells from different cell populations (Supplementary Note S2).

NDM has the same number of rows and columns as the original gene expression matrix (GEM) but reflects the importance of each gene in the network instead of the gene expression levels. This matrix can be analyzed by any traditional scRNA-seq algorithm for cell clustering, dimension-reduction and pseudo trajectory analysis by simply replacing the original GEM with our NDM, and thus our CSN method opens a new way to analyze scRNA-seq data at the network level. The input, output and application fields of our CSN method are listed in Supplementary Note S3.

### ‘Dark’ genes revealed by NDM

By NDM, we are able to reveal ‘dark’ genes, which have no significantly differential changes in terms of gene expression, and thus cannot be found by traditional differential analyses, but they are hub genes in the network or have significantly differential changes in terms of network degree, thereby may also play an important role in the network regulation.

### Clustering, dimension-reduction and pseudo trajectory analysis

One significant advantage of our method is that NDM can be further analyzed for clustering, dimension-reduction and pseudo-trajectory construction from a network perspective by any existing scRNA-seq method. In this paper, we select several existing methods that are widely used in scRNA-seq analysis to compare the performances of both NDM and original GEM. It should be noted that we focus not on the clustering methods themselves, but on the comparison between the traditional gene expression (GEM) and our network degree (NDM). Hence, in order to make it comparable, the parameters of all methods were set the same for GEM and NDM, and usually we used the default parameters, which are listed in Supplementary Note S4.

We used hierarchical clustering, *k*-means, *k*-medoids, SNN-Cliq and SIMLR to perform clustering analysis. Hierarchical clustering groups data by creating a cluster tree with multilevel hierarchy, where clusters at one level are joined as clusters at the next level. *k*-means (12) and *k*-medoids (13) clustering partition data into *k* mutually exclusive clusters, which minimize the sum of distances between an observation and its cluster center. In k-means, the cluster center is the mean of observations in this cluster. In *k*-medoids, the cluster center is a member of this cluster, called a medoid. SNN-Cliq (14) is a graph theory based clustering method, which utilizes the concept of shared nearest neighbor (SNN) to define cell similarity. SIMLR (8) is an analytic framework that learns a similarity measure from single-cell RNA-seq data based on multi-kernel learning. In this work, we also used hierarchical and *k*-means clustering to the data that is preprocessed by dimension-reduction of t-SNE (15). In hierarchical clustering, k-means, k-medoids and SIMLR, the number of clusters was set the same as the number of cell types, and SNN-Cliq used its internal algorithm to determine the number of clusters.

We used principal component analysis (PCA) and t-distributed stochastic neighbor embedding (t-SNE) (15) that represent linear and nonlinear methods to perform dimension-reduction analysis and visualization. Both of the methods have been widely used in the analysis of scRNA-seq data.

We used Wanderlust (16) in pseudo trajectory analysis, which constructs no-branch pseudo trajectory by giving each cell a value to represent the cell order, and the cells in the later stages will get larger values. In this work, we used the known time series of the dataset as gold standard. Compare every two cells’ Wanderlust values. If cell *i* is in the later stage of cell *j* and Wanderlust value of cell *i* is larger than cell *j, T* plus 1; if cell *i* is in the later stage of cell *j* but Wanderlust value of cell *i* is NOT larger than cell *j, F* plus 1. Then we used the value of *T* / (*T*+*F*) to measure the accuracy of the pseudo trajectory.

### Normalization and preprocessing methods

NDM comes straightforwardly from GEM (Figure 1), i.e. initial matrix → GEM → NDM. Generally, the normalization and preprocessing methods to the initial matrix will influence the performance of both GEM and NDM. The normalization methods are listed in Supplementary Note S5 for each dataset. In addition, we also compared the clustering performance of GEM and NDM from different normalization methods including FPKM, TPM and counts, which are the most widely-used in scRNA-seq studies.

The usual preprocessing methods to initial data matrix include gene selection and imputation. In this work, we only discarded the genes that were expressed in a small number of cells (<10 cells) or never expressed, and thus most genes (∼15 000 genes) were used. The number of genes used in GEM and NDM on each dataset is listed in Supplementary Note S5. In addition, we also compared the clustering performance of GEM and NDM based on different gene selection rules. Imputation was not used in the CSN and NDM construction, but as some studies (7) indicated that the imputation to the zero counts may solve the problem of dropout events in scRNA-seq data, we also compared the performance of GEM and NDM from the imputed data by scImpute (7). In addition, we took logarithm log(1+*x*) to the initial matrix, which is used in almost all scRNA-seq data.

### Comparison with bulk RNA-seq data

Besides single-cell RNA-seq data, our CSN method can be applied to the analysis of bulk RNA-seq data provided that there are a large number of samples which are required by our method. In this work, we conducted the CSN studies on TCGA adenocarcinoma and squamous cell carcinoma bulk RNA-seq data (Project: TCGA-LUAD and TCGA-LUSC, https://cancergenome.nih.gov) due to the large sample size, in the same way as scRNA-seq analysis.

### Datasets used for validation of CSN

In this work, we collected several high-quality datasets from the literatures to demonstrate the advantages of our CSN method. These datasets include human and mouse embryonic stem cells, cortical cells, tumor cells and so on, which represent various studies in scRNA-seq. The cell types in most datasets are quite clear as they are defined by the different cell sources (e.g. blood cells, neural cells), different cases (e.g. patients, normal people) or different time points. For other datasets, marker genes or FACS assay with respective markers were used to identify cell types, which also ensures the quality of data. Brief introductions and sources of all datasets are listed here and in Supplementary Note S5, respectively.

*Buettner dataset* (17) includes 182 cells and three cell types. This dataset contained mouse embryonic stem cells under different cell-cycle stages that have been annotated (G1, S and G2/M). 5600 genes are obtained per cell on average.

*Kolodziejczyk dataset* (18) includes 704 cells and three cell types. This dataset was obtained from a stem cell study on how different culture conditions influence pluripotent states of mouse embryonic stem cells. The cells came from several experiments involving three different culture conditions: serum + LIF, 2i + LIF and alternative 2i + LIF. 7700 genes are obtained per cell on average.

*Pollen dataset* (19) includes 249 cells and 11 cell types. This dataset includes skin cells, pluripotent stem cells, blood cells, neural cells and so on, which was designed to test the utility of low-coverage single-cell RNA-seq in identifying distinct cell populations. 7200 genes are obtained per cell on average.

*Zeisel dataset* (20) includes 3005 cells and nine cell types. This dataset contained the cells from the mouse cortex and hippocampus. The cell types including interneurons, S1 pyramidal cells, CA1 pyramidal, mural cells, endothelial cells, microglia, ependymal cells, astrocytes and oligodendrocytes were identified by hierarchical biclustering and validated by gene markers. 3700 genes are obtained per cell on average.

*Darmanis dataset* (21) includes 420 cells and eight cell types. This dataset contained the cells of human cortical tissue from eight adults and four embryonic samples. The cell types including OPCs, oligodendrocytes, astrocytes, microglia, neurons, endothelial cells, replicating neuronal progenitors and quiescent newly born neurons were identified by unbiased clustering and validated by some gene markers derived from the mouse brain. 4100 genes are obtained per cell on average.

*Chu-type dataset* (22) includes 1018 cells and seven cell types. This dataset contained the cells of human embryonic stem cell-derived lineage-specific progenitors. The cell types including H1 embryonic stem cells, H9 embryonic stem cells, human foreskin fibroblasts, neuronal progenitor cells, definitive endoderm cells, endothelial cells and trophoblast-like cells were identified by fluorescence-activated cell sorting (FACS) with their respective markers. 9600 genes are obtained per cell on average.

*Chu-time dataset* (22) includes 758 cells and six cell types. This dataset contained the cells with 6 time points along the differentiation protocol to produce definitive endoderm cells from human ES cells. A total of 758 cells were captured and profiled by scRNA-seq at 0, 12, 24, 36, 72 and 96 h of differentiation. 8700 genes are obtained per cell on average.

*Kim dataset* (23) includes 118 cells and three cell types. This dataset is designed to identify successful clonal propagation from patient to PDX samples and understand pathogenesis from primary (pRCC) to metastatic renal cell carcinoma (mRCC). The cell types including the tumor cells from the parental mRCC, PDX-mRCC and PDX-pRCC were identified by fluorescent microscopic observation. 6700 genes are obtained per cell on average.

*Trapnell dataset* (24) includes 372 cells and four cell types. This dataset contained the cells with four time points along the differentiation protocol of primary human myoblasts. Cells were first cultured in high-serum medium, and then, after a switch to low-serum medium, cells were dissociated, individually captured and profiled by scRNA-seq at 0, 24, 48 and 72 h. 6600 genes are obtained per cell on average.

*Xin dataset* (25) includes 1600 cells and four cell types. This dataset contained the human pancreatic α-, β-, δ- and PP cells from non-diabetic and type 2 diabetes organ donors. 5700 genes are obtained per cell on average.

*TCGA lung cancer data* (Project: TCGA-LUAD and TCGA-LUSC) is bulk RNA-seq dataset, including 1135 samples from 1016 cases, which comprise of 524 adenocarcinoma samples, 61 adenocarcinoma adjacent normal tissues samples, 501 squamous cell carcinoma samples and 49 squamous cell carcinoma adjacent normal tissues samples. In this work, we used the dataset normalized by FPKM and selected 33 409 genes that are expressed in at least 500 samples to calculate GEM and NDM.

## RESULTS

### Network analysis on a single-cell basis

**Figure 3.**
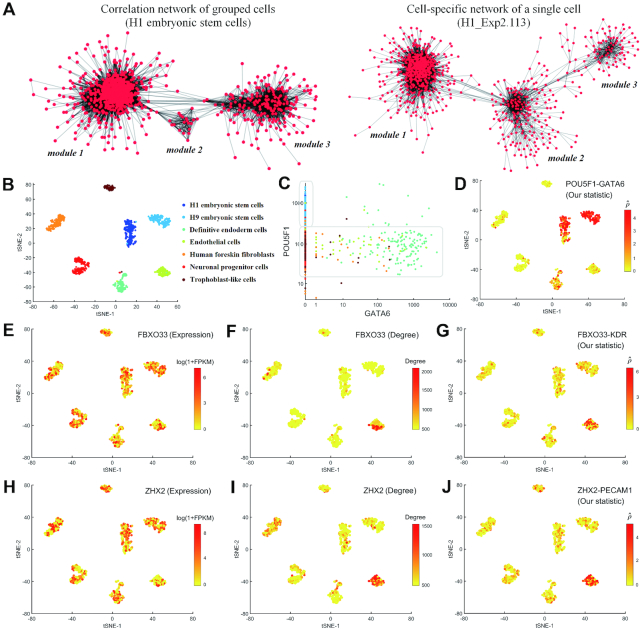
Illustration of network analyses of *Chu-type* dataset based on our CSN method. (**A**) Correlation network of grouped cells (edge means correlation coefficient of two genes > 0.7) and CSN of a single cell (edge means normalized statistic of two genes > 8). The genes used in the network construction are all the same. (**B**) t-SNE plots of *Chu-type* dataset, where different cell types could be distinguished clearly (ARI = 0.98). (**C**) Scatter diagrams of genes POU5F1 and GATA6, colored by the cell types listed in (B). (**D**) Performance of edge POU5F1 - GATA6 in the t-SNE plots, colored by the normalized statistic. (**E**-**J**) Performance of genes FBXO33 and ZHX2 in the t-SNE plots, colored by (**E**) the gene expression level of FBXO33, (**F**) the network degree level of FBXO33, (**G**) the normalized statistic of edge FBXO33-KDR, (**H**) the gene expression level of ZHX2, (**I**) the network degree level of ZHX2, (**J**) the normalized statistic of edge ZHX2-PECAM1.

In this paper, we performed network analysis based on our CSN method to *Chu-type dataset* (22), which was illustrated in Figure 3. This dataset was obtained from a study of developmental biology, which contains seven cell types including H1 embryonic stem cells (H1), H9 embryonic stem cells (H9), human foreskin fibroblasts (HFF), neuronal progenitor cells (NPC), definitive endoderm cells (DEC), endothelial cells (EC) and trophoblast-like cells (TB). These cell types can be distinguished clearly by t-SNE in both GEM and NDM. Figure 3A illustrates the genes correlation networks constructed by the grouped cells (H1) and by the CSN of a single cell (H1_Exp2.113, GEO sample ID: GSM1966635), respectively. It is obvious that in spite of some differences, both networks comprise of three modules, where most genes from the two networks are present in the similar way (in particular, in module 1). Figure S7 (Supplementary Note S6) also illustrates the high relevance between the correlation coefficient and normalized statistic of two genes. Thus, the CSN of each cell is generally similar to the correlation network constructed by the grouped cells, and any tool of network studies can be also used for the further analysis of the constructed CSNs.

However, CSN is a method for a single-cell network, which makes it possible to analyze the gene–gene associations at a single-cell resolution. Figure 3C illustrates the scatter diagram of genes POU5F1 and GATA6. We can see the high level of POU5F1 corresponds to the zero expression of GATA6, which is in agreement with the experimental results that POU5F1 inhibits the expression of GATA6 and down regulation of POU5F1 is accompanied by the increased expression of the endoderm-associated genes GATA6 in human embryonic stem cells (26,27). We can also see the low level of POU5F1 cannot inhibit the expression of GATA6 and there is little association between GATA6 and the low level of POU5F1. From the performance of the normalized statistic of edge POU5F1-GATA6 based on CSN method (Figure 3D), we can see clearly that POU5F1 and GATA6 are associated in H1 and H9 embryonic stem cells where POU5F1 is highly expressed, and not associated in the other cell types where POU5F1 is lowly expressed. By contrast, if we construct the correlation networks for each cell type based on the grouped cells instead of single cell (i.e. one network for one cell type), we will find the correlations between POU5F1 and GATA6 are always almost zero. Moreover, if we estimate the correlation based on all cells, we can find the global negative correlation but do not know in which cell types the two genes are associated. This is the limitation of the networks constructed by the grouped cells, and could be overcome by CSN method (Supplementary Note S7).

From Figure 3D, we can also see the values of the normalized statistic are significantly high in some cell types, which indicates that there are some strong associations between the two genes specifically in these cell types. Based on the statistic test, we can find the differential edges just similarly as the differential genes, and thus, not only key genes, but also key gene-associations can be identified, though these associations are not necessarily direct or causal relations.

In addition, many important genes interact with multiple partners and thus our CSN method is able to find key genes from a network viewpoint, e.g. the hub genes with high degrees. Supplementary Note S8 lists the top 10 genes of each cell type on *Chu-type dataset* with the highest degree. We can see that some genes such as POU5F1, L1TD1 and PCGF1 have been validated to play a key role in cell differentiation and pluripotency. POU5F1 encodes a transcription factor that plays an important role in embryonic development and stem cell pluripotency (28). LITD1 is related to the post-transcriptional regulation in human pluripotency (29). PCGF1 represents a physical and functional link between Polycomb function and pluripotency (30).

### ‘Dark’ genes

Based on CSN method, we can not only find the differential expression genes but also the differential degree genes. If a gene has a significant difference between case and control samples not in a gene expression level but in a network degree level, we call this gene as ‘dark’ gene (Supplementary Note S9). Figure 3E–J illustrate some ‘dark’ genes of endothelial cells (EC). It is obvious that genes FBXO33 and ZHX2 show high degrees in EC (low degrees in other cell types), but at the expression level, there is no significant difference among all cell types. We can see the obvious differential associations between FBXO33 and KDR, and between ZHX2 and PECAM1. In fact, KDR encodes one of the two receptors of vascular endothelial growth factor (VGCF), and VGCF is a major growth factor for endothelial cells. The protein encoded by PECAM1 makes up a large portion of endothelial cell intercellular junctions (NCBI Gene, https://www.ncbi.nlm.nih.gov/gene). These results imply FBXO33 and ZHX2 may also play biological roles in EC, which needs to be further studied in future.

### Network rewiring on a single-cell basis

**Figure 4.**
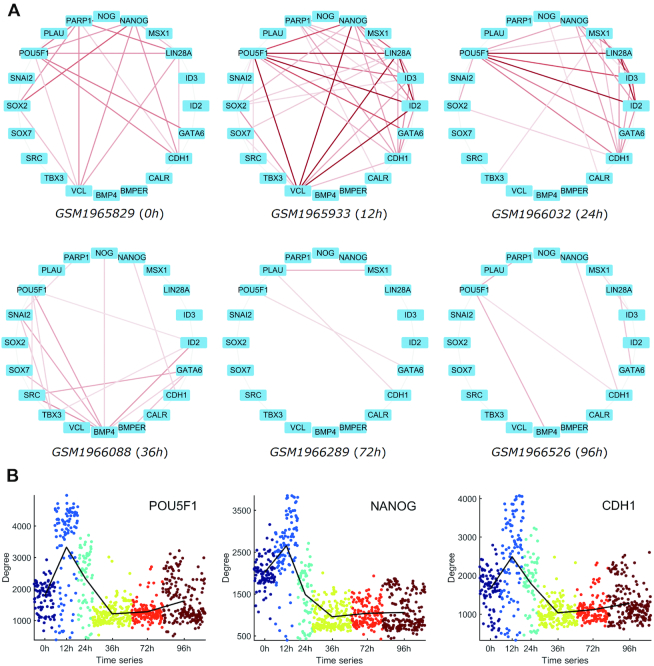
Illustration of network analyses of *Chu-time* dataset based on our CSN method. (**A**) CSNs of some single cells with the 20 genes that are involved in human embryo development, where the larger normalized statistic leads to the darker edge. (**B**) Network degrees of POU5F1, NANOG and CDH1 along the six time points of embryo development.

In this paper, we also performed the network rewiring analysis on *Chu-time dataset* (22), which came from a study of developmental biology, and contained 758 cells with 6 time points (0, 12, 24, 36, 72, 96 h) along the differentiation protocol to produce definitive endoderm cells from human embryonic stem cells. Figure 4A illustrates the partial CSNs of some single cells with the 20 genes that are involved in human embryo development. We can see the network topology changes dynamically at different time points. At 12 h, the associations among these genes are the strongest, while at 72 h and 96 h, the associations become quite weak. We can also see the network degrees of POU5F1, NANOG and CDH1 show their peaks at 12 h from Figure 4B, which means these genes are correlated with more other genes and may play important roles as hub genes from a network viewpoint at 12 h. These results imply that the key time point may be around 12 h due to the drastic network rewiring during the embryo development.

### Network-based cell clustering and gene dimension-reduction analysis

**Figure 5.**
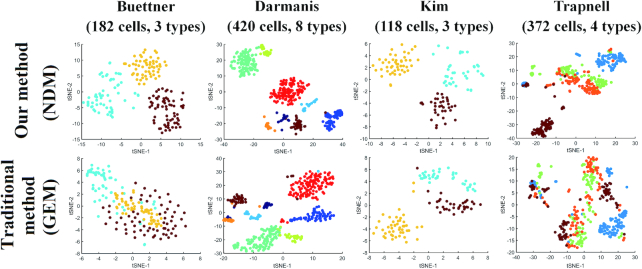
The clustering performances of NDM and GEM on four datasets. t-SNE plots are used for visualization and different colors represent different cell types.

Based on our CSN method, we used several algorithms including hierarchical clustering, *k*-means (12), *k*-medoids (13), SNN-Cliq (14) and SIMLR (8) to perform the cell clustering analysis, PCA and t-SNE (15) that represent linear and nonlinear methods to perform dimension-reduction analysis. GEM and NDM were used for comparison on the nine datasets from literatures (17–24), where we adopted the same the algorithm parameters (Supplementary Note S4) and normalization method (Supplementary Note S5). As the classification label of each observation had been known, adjusted random index (ARI), F1-measure, purity and entropy were used as the indexes in comparison. From the results shown in Table 1 and Supplementary Note S10, we can see the superior performances of our NDM over the original GEM for various methods clearly. The best results of all datasets come from our NDM and even linear method such as hierarchical clustering can produce quite good performances based on our NDM. In the dimension-reduction analysis shown in Figure 5 and Supplementary Note S11, NDM can also distinguish different cell types more clearly than GEM by both linear (PCA) and nonlinear (t-SNE) method.

**Table 1.** The comparison of GEM and NDM in clustering analysis, evaluated by adjusted random index (ARI)

		Buettner	Kolod ziejczyk	Pollen	Zeisel	Darmanis	Chu-type	Chu-time	Kim	Trapnell
Hierarchical	GEM	0.48	0.49	0.95	0.55	0.63	0.75	0.67	0.66	0.08
	**NDM**	**0.82**	**0.99**	**0.96**	0.53	**0.91**	**0.77**	**0.72**	**0.73**	**0.24**
*k*-means	GEM	0.31	0.53	0.90	0.39	0.58	0.73	0.59	0.60	0.14
	**NDM**	**0.74**	**0.80**	0.87	**0.43**	**0.77**	**0.77**	**0.70**	**0.83**	**0.44**
Hierarchical (tSNE)	GEM	0.32	0.99	0.94	0.60	0.67	0.98	0.68	0.66	0.16
	**NDM**	**0.97**	**1.00**	0.85	**0.62**	**0.86**	**0.99**	**0.68**	**1.00**	**0.43**
*k*-means (tSNE)	GEM	0.30	0.99	0.94	0.62	0.65	0.98	0.69	0.72	0.16
	**NDM**	**0.94**	**1.00**	0.85	**0.65**	**0.85**	**0.99**	**0.69**	**1.00**	**0.47**
*k*-medoids	GEM	0.14	0.03	0.91	0.43	0.36	0.60	0.43	0.57	0.00
	**NDM**	**0.31**	**0.73**	0.89	0.11	0.23	**0.76**	0.41	**0.61**	**0.23**
SIMLR	GEM	0.92	0.99	0.90	0.56	0.75	0.74	0.66	0.97	0.21
	**NDM**	**1.00**	**1.00**	**0.92**	**0.67**	**0.90**	**0.75**	**0.67**	0.95	**0.31**
SNN-Cliq	GEM	0.00	0.00	0.90	0.50	0.20	0.64	0.30	0.58	0.00
	**NDM**	**0.50**	**0.65**	**0.90**	**0.60**	0.01	0.61	**0.36**	**0.58**	**0.24**

Hierarchical (tSNE) and *k*-means (tSNE) represent that the clustering analysis is performed after dimension-reduction by t-SNE.

### Network-based cell pseudo trajectory analysis

In this work, we used two datasets with the gold standard from literatures to perform pseudo trajectory analysis, which include 758 cells with 6 stages (0, 12, 24, 36, 72, 96 h) in *Chu-time* dataset (22) and 372 cells with four stages (0, 24, 48, 72 h) in *Trapnell* dataset (24). Wanderlust (16) is a method to construct no-branch pseudo trajectory and GEM and NDM are used for comparison. From the results shown in Figure S10 (Supplementary Note S12), we can see the Wanderlust values increase in accordance with the time sequence in *Chu-time* dataset, and the results of GEM and NDM are quite similar, whose accuracy is 0.92 and 0.93 respectively. But in *Trapnell* dataset, NDM is able to identify the change at 72 h, but GEM fails, and the accuracy of GEM and NDM is 0.62 and 0.73 respectively. Thus, it is indicated that NDM is also able to reconstruct the time series of single cells corresponding to the developmental stages, and may produce similar or better results than the original GEM.

### Comparison of NDM for different CSN parameters, normalization and preprocessing methods

In this paper, we set *n_x_*^(^*^k^*^)^ = *n_y_*^(^*^k^*^)^ = 0.1*n*, where the coefficient 0.1 is called as the box size. Figure S11 (Supplementary Note S13) illustrates how the ARI in clustering analysis changes with the box size and *P*-value in different datasets. It is indicated that the optimum box size is about 0.1, and the optimum *P*-value is about 0.01 on average, which are set as the default parameters of CSN method.

We compared different normalization methods on the same dataset in Supplementary Note S14. We can see NDM from the GEM normalized by TPM/FPKM/count gets the similar performances on the same dataset, though the result by TPM seems to be better. Thus, our NDM method is not sensitive to the normalization method, and is suitable to various types of gene expression matrix.

We also compared different gene selection rules on the same dataset in Supplementary Note S15. We can see the different gene selection rules such as ‘FPKM per cell on average >1 (or >5, >10, >50)’ have just a little influence on the performance of GEM and NDM. Clearly, NDM is still superior to GEM and is also not so sensitive to the gene selection rules.

Imputation was not used in the construction of CSN and NDM in this paper except Supplementary Note S16 that uses the imputation for comparison purpose. We compared the clustering performance of GEM and NDM from the imputed data in Figure S12 (Supplementary Note S16). We can see that the imputed GEM gets better results than the original GEM in some datasets, but is usually inferior to the performance of NDM from the original GEM. The result of NDM from the imputed GEM is slightly better than the imputed GEM, but is obviously worse than NDM from the original GEM. As a conclusion, the imputation by existing methods is not recommended in our CSN construction. Existing imputation methods such as scImpute (7) are based on the expression level of scRNA-seq data without the consideration of the gene associations, and thus may result in the alterations of edges or correlations between genes, which leads to the worse performance of the NDM from the imputed data for some cases.

### Comparison with bulk RNA-seq data

**Figure 6.**
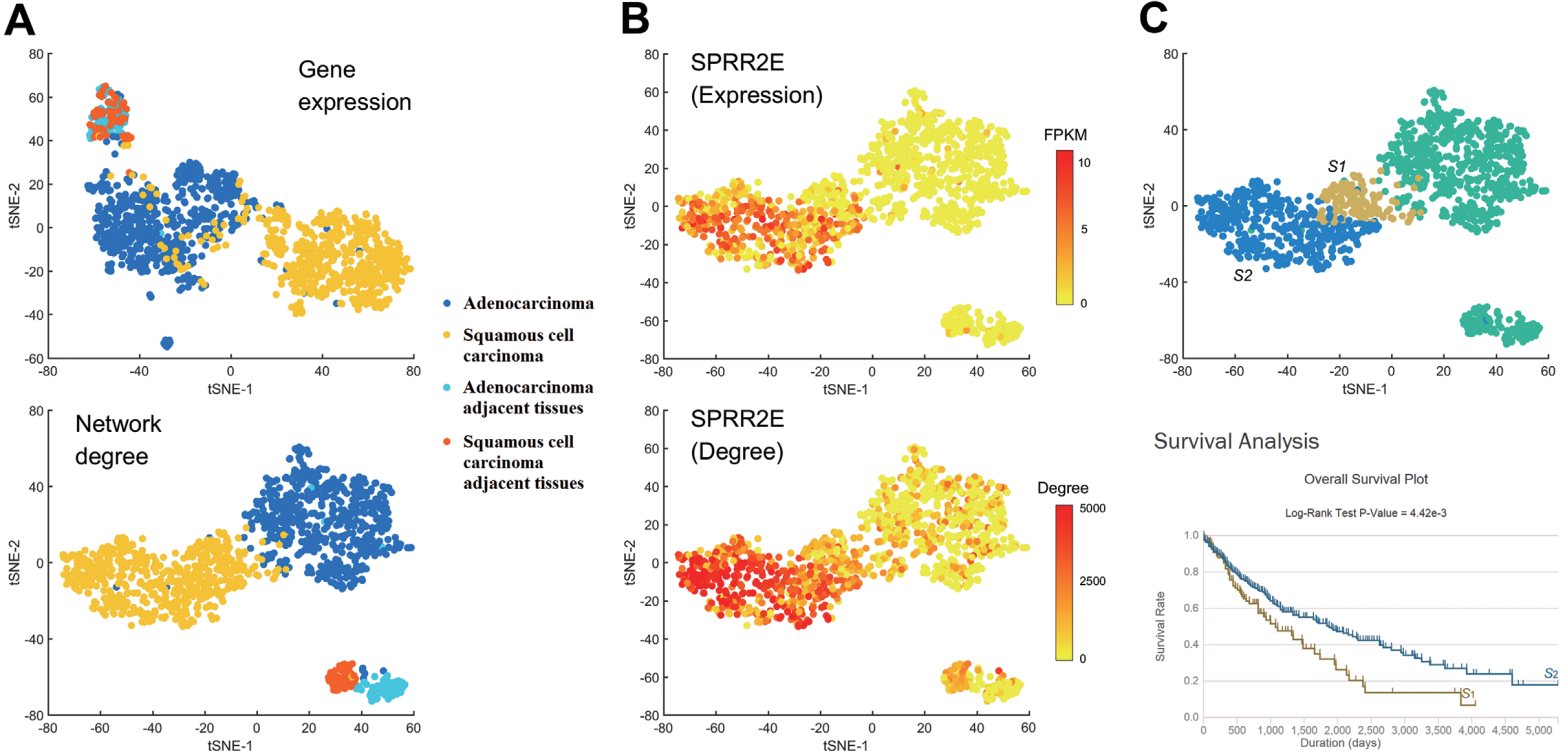
Comparison of GEM and NDM based on TCGA adenocarcinoma and squamous cell carcinoma bulk RNA-seq data. (**A**) Clustering performance (t-SNE) between GEM and NDM. Different colors represent different cell types. (**B**) The expression level FPKM and network degree of gene SPRR2E. (**C**) Squamous cell carcinoma can be divided into two parts S1 and S2 based on NDM, and the survival analysis shows the significant difference between S1 and S2.

In this work, we also applied our CSN method to TCGA lung cancer bulk RNA-seq data. Based on the clustering analysis shown in Figure 6A, we can see the distinctions among adenocarcinoma (AD), squamous cell carcinoma (SC) and adjacent normal tissues are clearer based on NDM than GEM (ARI of GEM = 0.69 and ARI of NDM = 0.81, t-SNE + *k*-means clustering), and especially, clustering analysis based on NDM is able to distinguish AD adjacent tissues and SC adjacent tissues, while GEM fails. We can also find some genes that show significant difference between the two types of adjacent tissues in terms of both expression and network degree (Supplementary Note S17), but some genes such as SPRR2E only show the difference in terms of network degree (Figure 6B). This result implies that AD adjacent tissues and SC adjacent tissues are different, and their major differences can be revealed in the gene associations or at a network level instead of gene expression. In addition, we can divide SC samples into two parts based on NDM (Supplementary Note S18), and the survival analysis shows the significant difference between the two parts (Figure 6C), which validated the effectiveness of our CSN method for the identification of possible new subtype of lung cancers. Thus, our method is also suitable for bulk RNA-seq data if the sample size is large, and NDM shows obvious advantage over traditional GEM in clustering analysis. In addition, our NDM may find some new subtypes of cancers, which has potential applications in medicine or personalized treatment.

Moreover, we identified a number of the ‘dark’ genes in TCGA lung cancer bulk RNA-seq data. Those ‘dark’ genes have no differential expressions between lung cancer samples and normal samples, which are ignored by the traditional methods. But by our CSN method, they are found to have significantly differential network degrees, which are considered important at a network level. Actually, although those ‘dark’ genes by their gene expressions cannot prognose the lung cancer samples, survival analyses on those ‘dark’ genes from TCGA clinic data validated that they can be used to make the prognosis analysis or prediction on the lung cancer samples by their network degrees, and thus may have potential applications in medicine (Supplementary Note S19).

## DISCUSSION

CSN provides a method to analyze gene associations at a single-cell level, and thus we can find differential gene associations just similar as differential genes. Gene regulations are essential for many important biological processes such as transcriptional regulation, co-expression, alternative splicing, DNA modification and function of non-coding RNA, and are presented as the dependency between two genes in the scRNA-seq data. For example, if genes *x* and *y* are co-expressed, the RNA levels of the two genes will be positively correlated, and if *x* and *y* are different alternatively spliced transcripts from the same pre-mRNA, the RNA levels of the two genes will be usually negative correlated. Our CSN method is able to identify the dependency and independency of two genes in a single cell, and then finds the changes of gene associations among different cell types. In addition, as an unsupervised method, CSNs are directly constructed from the gene expression matrix without the pre-knowledge on clusters or cell types, and thus the analysis based on CSN is unbiased.

A biological process can be viewed as the evolution of a dynamical system with gene/protein as variables, it can be represented as $\dot{x}$(*t*) = *f* (*x* (*t*)), where *x*(*t*) is gene expressions or molecular concentrations changing dynamically or even drastically with the time and conditions, and *f* is the functions or linear/nonlinear associations among genes which generally remain unchanged or change gradually with small perturbations (31–36). Thus, gene expressions are considered too ‘volatile/unstable’ to characterize the status of the biological process, comparing to the gene associations which are ‘stable’ features. Though the variance of scRNA-seq reveals the heterogeneity and functional diversity among cell population, the wide variance will interfere with the distinguishing of key genes in different cell types or developmental stages. In contrast, the gene associations are stable with small perturbations, and thus reliable to characterize the cell types or clusters, and also key genes. Our CSN method can be viewed as the data transformation from ‘unstable’ gene expression form to ‘stable’ gene association form on a single-cell basis. Thus, rather than the originally measured GEM data, we use the transformed NDM for further analysis, which can reliably characterize the cell states. From the results, regardless of the analysis approaches, our NDM illustrated better performance than original GEM on most datasets. The network degree is able to distinguish different cell types and reconstruct the time series of single cells corresponding to the developmental stages, and each of cell type or developmental stage has similar gene associations rather than similar gene expressions. The result validated the effectiveness of CSN, and demonstrated that it is the gene associations that stably characterize the cell types or developmental process.

Traditionally, we use differential expression analysis to find the important genes, but small changes of some genes may lead to a large biological effect, which makes this kind of key genes ignored by traditional analysis. In this paper, CSN method measures the biological effect of each gene from the network perspective, and may identify these ‘dark’ genes even with significant difference between case and control samples not in a gene expression level but in a network degree level.

**Figure 7.**
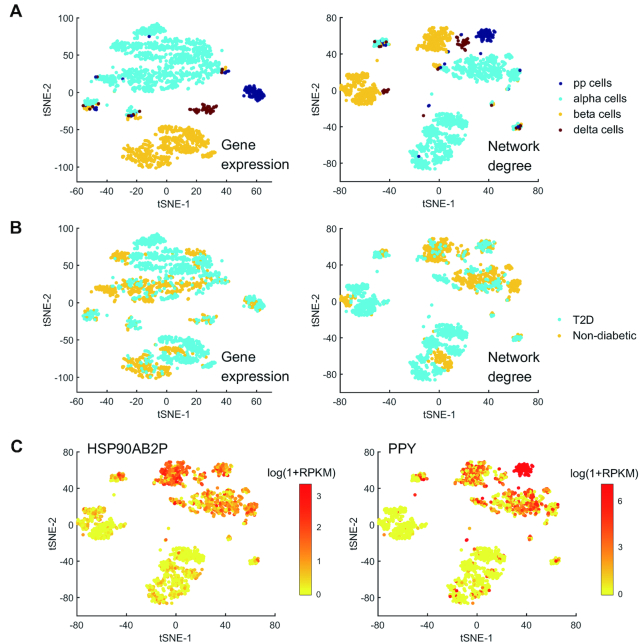
Cell type analysis of *Xin dataset*. (**A**) Clustering performance (t-SNE) of *Xin dataset* based on GEM and NDM. Different colors represent different cell types (pp cells, alpha cells, beta cells and delta cells). (**B**) Clustering performance of *Xin dataset* based on GEM and NDM. Different colors represent different cell sources (T2D and non-diabetic). (**C**) The expression levels of genes HSP90AB2P and PPY.

In biology, cell types are defined by morphology and functionality. As different cell types usually exhibit distinct transcriptional expression patterns, scRNA-seq may help to detect new cell types based on the clustering analysis of gene expression data. In this work, we provided a new method based on the network degree data, which is a valuable supplement to the traditional methods. Figure 7 illustrates the clustering of *Xin dataset* (25) that contains 1600 human pancreatic α-, β-, δ- and PP cells from non-diabetic and type 2 diabetes (T2D) organ donors. Based on the clustering result of GEM, we can see the four cell types can be distinguished clearly, but it is hard to distinguish the cells of non-diabetic donors from T2D donors. This result is in agreement with the literature (25). By contrast, in the result of NDM, the four cell types can be also distinguished, but α-, β- and δ-cells are obviously divided into two parts. Furthermore, we can find some genes that are significantly different in expression between the two parts (Figure 7B), which implies that the human pancreatic α-, β- and δ-cells may be further divided into two subtypes. Of course, the division of clusters may also come from the different experimental conditions instead of different cell types, and thus scRNA-seq alone cannot define new cell types and further validation by experiments is essential.

In addition, our method can be also applied to bulk RNA-seq datasets for constructing individual network of each single sample in a similar way provided that there are a large number of samples, which indicates the wide applications to network biology. However, there are still some limitations in our CSN method. CSN is a kind of correlation network instead of causal network. Hence, the identified associations are not necessarily causal relations between two genes, which is actually one of our future topics.

## DATA AVAILABILITY

The datasets supporting the conclusions of this article are listed in Supplementary Note 5. The source code is available in Supplementary Note 20 and Hao Dai's GitHub repository (https://github.com/wys8c764/CSN).

## Supplementary Material

gkz172_Supplemental_File
