# Supplementary material for: Cell-specific network constructed by single-cell RNA sequencing data
Source: Nucleic Acids Res. 2019 Mar 13;47(11):e62. doi: 10.1093/nar/gkz172 (PMC6582408; doi:10.1093/nar/gkz172)
Supplement: gkz172_Supplemental_File [file gkz172_supplemental_file.pdf]

Cell-specific Network Constructed by Single-cell RNA Sequencing Data

Supplementary Data

Hao Dai[1], Lin Li[1], Tao Zeng[1] and Luonan Chen[1,2,3,4,*]

[1] Center for Excellence in Molecular Cell Science, Institute of Biochemistry and Cell Biology, Chinese Academy of Sciences, Shanghai 200031, China

[2] Center for Excellence in Animal Evolution and Genetics, Chinese Academy of Sciences, Kunming 650223, China

[3] School of Life Science and Technology, ShanghaiTech University, Shanghai 200031, China

[4] Shanghai Research Center for Brain Science and Brain-Inspired Intelligence, Shanghai 201210, China

* Address correspondence to this author, Institute of Biochemistry and Cell Biology, Chinese Academy of Sciences, Yueyang Road 320, Shanghai 200031, China; Email: lnchen@sibs.ac.cn

Supplementary Note 1: Statistic of cell-specific network

(1) Statistic

Assume that there are n cells with m genes in scRNA-seq data. Based on the statistical independency in probability theory (eqn. (1) in the main text), we design a statistic for each gene pair and each cell as

$$\rho_{xy}{}^{(k)} = \frac{n_{xy}{}^{(k)}}{n} - \frac{n_x{}^{(k)}}{n} \cdot \frac{n_y{}^{(k)}}{n} \tag{S-1}$$

where $\rho_{xy}{}^{(k)}$ is the statistic for genes x, y of cell k (the red plot in Figure S1(A)), $n_x{}^{(k)}$ and $n_y{}^{(k)}$ are the number of plots (or cells) in the neighborhood of x_k and y_k respectively (light and medium grey boxes in Figure S1(A)), $n_{xy}{}^{(k)}$ is the number of plots in the neighborhood of (x_k, y_k) (intersection of two boxes and represented as dark grey box in Figure S1(A)), and n is the total number of plots.

In eqn. (S-1), $n_x{}^{(k)}$ and $n_y{}^{(k)}$ are determined in advance ($< n$), and thus this statistic is only changed with $n_{xy}{}^{(k)}$. Hence, as shown in Figure S1 (A), we first draw the two boxes $D_x{}^{(k)}$ and $D_y{}^{(k)}$ to represent the neighborhood of x_k and y_k respectively based on the predetermined $n_x{}^{(k)}$ and $n_y{}^{(k)}$, and then we can straightforwardly have the third box $D_{xy}{}^{(k)}$, which is simply the intersection of the previous two boxes. By counting the plots in $D_{xy}{}^{(k)}$, we can obtain the value of $n_{xy}{}^{(k)}$ and then get the criterion of eqn. (S-1), as shown in Figure S1(B).

(A)

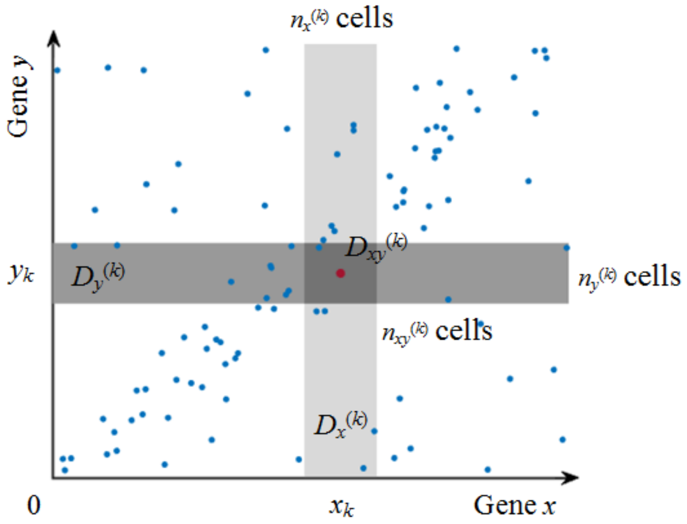

(B)

$$\rho_{xy}{}^{(k)} = \frac{n_{xy}{}^{(k)}}{n} - \frac{n_x{}^{(k)}}{n} \cdot \frac{n_y{}^{(k)}}{n} \leq 0 \quad \text{for}$$

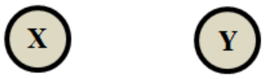

$$\rho_{xy}{}^{(k)} = \frac{n_{xy}{}^{(k)}}{n} - \frac{n_x{}^{(k)}}{n} \cdot \frac{n_y{}^{(k)}}{n} > 0 \quad \text{for}$$

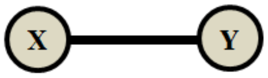

Figure S1. Scatter diagram of the expression values of gene x, y for cell k, and illustration of the three boxes for our statistic. (A) Near the red plot k, make the light and medium grey box $D_x{}^{(k)}$ and $D_y{}^{(k)}$ to represent the neighborhood of x_k and y_k respectively. The number of plots in the two boxes are $n_x{}^{(k)}$ and $n_y{}^{(k)}$, which are predetermined ($< n$). The intersection of the two boxes is the dark grey box $D_{xy}{}^{(k)}$ that represents the neighborhood of (x_k , y_k), in which the number of plots is $n_{xy}{}^{(k)}$. (B) Criterion based on the

statistic $\rho_{xy}^{(k)}$. We can identify the association of two genes by statistical test based on the statistic $\rho_{xy}^{(k)}$.

Note that the distribution of genes has no influence on the statistic. The box size of $D_x^{(k)}$ and $D_y^{(k)}$ will change with the density of plots, smaller in the dense area and larger in the sparse area. Then, no matter which distribution the genes follow, the boxes in Figure S1 cover the same plots and the statistic model will not change.

(2) Mean value and variance of the statistic

As $n_x^{(k)}$ and $n_y^{(k)}$ are predetermined and $n_{xy}^{(k)}$ follows binomial distribution, based on Bayes formula, we can get

$$P\left(n_{xy}^{(k)} = t \middle| n_x^{(k)} = i, n_y^{(k)} = j\right)$$

$$= \frac{P\left(n_{xy}^{(k)} = t \middle| n_x^{(k)} = i\right) P\left(n_{xy}^{(k)} = t \middle| n_y^{(k)} = j\right)}{P\left(n_{xy}^{(k)} = t\right)}$$

$$= \frac{\frac{i!}{t!\,(i-t)!} \cdot \left(\frac{p_{xy}}{p_x}\right)^t \left(1 - \frac{p_{xy}}{p_x}\right)^{i-t} \cdot \frac{j!}{t!\,(j-t)!} \cdot \left(\frac{p_{xy}}{p_y}\right)^t \left(1 - \frac{p_{xy}}{p_y}\right)^{j-t}}{\frac{n!}{t!\,(n-t)!} \cdot p_{xy}{}^t \left(1 - p_{xy}\right)^{n-t}}$$

where p_x, p_y and p_{xy} is the probability that a plot is located in the $D_x^{(k)}$, $D_y^{(k)}$ and $D_{xy}^{(k)}$ respectively. Then

$$\sum_{t=0}^{n} P\left(n_{xy}^{(k)} = t \middle| n_x^{(k)} = i, n_y^{(k)} = j\right) = 1$$

$$\sum_{t=0}^{n} t \cdot P\left(n_{xy}^{(k)} = t \middle| n_x^{(k)} = i, n_y^{(k)} = j\right)$$

$$= \sum_{t=0}^{n} t \frac{\left[\frac{ip_{xy}}{tp_x} \cdot \frac{(i-1)!}{(t-1)!\,(i-t)!} \left(\frac{p_{xy}}{p_x}\right)^{t-1} \left(1 - \frac{p_{xy}}{p_x}\right)^{i-t}\right] \left[\frac{jp_{xy}}{tp_y} \cdot \frac{(j-1)!}{(t-1)!\,(j-t)!} \left(\frac{p_{xy}}{p_y}\right)^{t-1} \left(1 - \frac{p_{xy}}{p_y}\right)^{j-t}\right]}{\frac{np_{xy}}{t} \cdot \frac{(n-1)!}{(t-1)!\,(n-t)!} \cdot p_{xy}{}^{t-1} \left(1 - p_{xy}\right)^{n-t}}$$

$$= \frac{ij}{n} \cdot \frac{p_{xy}}{p_x p_y} \cdot \sum_{t=1}^{n} P\left(n_{xy}^{(k)} = t-1 \middle| n_x^{(k)} = i-1, n_y^{(k)} = j-1\right) = \frac{ij}{n} \cdot \frac{p_{xy}}{p_x p_y}$$

By the similar calculation, we can get

$$\sum_{t=0}^{n} t^2 \cdot P\left(n_{xy}^{(k)} = t \middle| n_x^{(k)} = i, n_y^{(k)} = j\right) = \frac{ij(i-1)(j-1)}{n(n-1)} \cdot \frac{p_{xy}{}^2}{p_x{}^2 p_y{}^2} + \frac{ij}{n} \cdot \frac{p_{xy}}{p_x p_y}$$

Then the mean value of the statistic is

$$\mu_{xy}^{(k)} = E\left(\frac{n_{xy}^{(k)}}{n} - \frac{n_x^{(k)} n_y^{(k)}}{n^2}\right) = \sum_{t=0}^{n} \left(\frac{t}{n} - \frac{ij}{n^2}\right) \cdot P\left(n_{xy}^{(k)} = t \middle| n_x^{(k)} = i, n_y^{(k)} = j\right)$$

$$= \frac{ij}{n^2} \left(\frac{p_{xy}}{p_x p_y} - 1\right)$$

The variance of the statistic is

$$\sigma_{xy}{}^{(k)2} = E\left[\left(\frac{n_{xy}{}^{(k)}}{n} - \frac{n_x{}^{(k)}n_y{}^{(k)}}{n^2}\right)^2\right] - \left[E\left(\frac{n_{xy}{}^{(k)}}{n} - \frac{n_x{}^{(k)}n_y{}^{(k)}}{n^2}\right)\right]^2$$

$$= \sum_{t=0}^{n}\left(\frac{t}{n} - \frac{ij}{n^2}\right)^2 \cdot P\left(n_{xy}{}^{(k)} = t \middle| n_x{}^{(k)} = i, n_y{}^{(k)} = j\right) - \left[\frac{ij}{n^2}\left(\frac{p_{xy}}{p_x p_y} - 1\right)\right]^2$$

$$= \frac{ij}{n^4} \cdot \frac{p_{xy}}{p_x p_y}\left[\frac{(n-i)(n-j)}{n-1} \cdot \frac{p_{xy}}{p_x p_y} + n\left(1 - \frac{p_{xy}}{p_x p_y}\right)\right]$$

If genes x and y are independent of each other, $p_{xy} = p_x p_y$, and thus

$$\mu_{xy}{}^{(k)} = 0$$

$$\sigma_{xy}{}^{(k)2} = \frac{n_x{}^{(k)}n_y{}^{(k)}\left(n - n_x{}^{(k)}\right)\left(n - n_y{}^{(k)}\right)}{n^4(n-1)}$$

We normalize the statistic $\rho_{xy}{}^{(k)}$ and define

$$\hat{\rho}_{xy}{}^{(k)} = \frac{\left(\frac{n_{xy}{}^{(k)}}{n} - \frac{n_x{}^{(k)}n_y{}^{(k)}}{n^2}\right) - 0}{\sqrt{\frac{n_x{}^{(k)}n_y{}^{(k)}\left(n - n_x{}^{(k)}\right)\left(n - n_y{}^{(k)}\right)}{n^4(n-1)}}} = \frac{\sqrt{n-1} \cdot \left(n \cdot n_{xy}{}^{(k)} - n_x{}^{(k)}n_y{}^{(k)}\right)}{\sqrt{n_x{}^{(k)}n_y{}^{(k)}\left(n - n_x{}^{(k)}\right)\left(n - n_y{}^{(k)}\right)}} \quad \text{(S-2)}$$

Eqn. (S-2) is the statistic model in this paper, or called the normalized statistic. If genes x and y are independent of each other, the mean value and variance of $\hat{\rho}_{xy}{}^{(k)}$ for the n cells are 0 and 1 respectively.

(3) Distribution of the statistic

By the similar derivation, we can get the third-order and fourth-order moments of the statistic. If genes x and y are independent of each other, $n_x^{(k)}$ and $n_y^{(k)}$ increase in proportion to n, and the third-order and fourth-order moments of the normalized statistic will tend to those of the standard normal distribution (0 and 3 respectively) with the increase of n.

$$\lim_{n\to\infty} E\left[\left(\hat{\rho}_{xy}{}^{(k)}\right)^3\right] = \lim_{n\to\infty} \frac{\sqrt{n-1}}{n-2} \cdot \frac{(n - 2n_x{}^{(k)})(n - 2n_y{}^{(k)})}{\sqrt{n_x{}^{(k)}n_y{}^{(k)}(n - n_x{}^{(k)})(n - n_y{}^{(k)})}} = 0$$

$$\lim_{n\to\infty} E\left[\left(\hat{\rho}_{xy}{}^{(k)}\right)^4\right] = \lim_{n\to\infty} \frac{n-1}{(n-2)(n-3)}\left[3(n+6) - \frac{6n^2}{n_x{}^{(k)}(n - n_x{}^{(k)})} - \frac{6n^2}{n_y{}^{(k)}(n - n_y{}^{(k)})} + \frac{n^3(n+1)}{n_x{}^{(k)}n_y{}^{(k)}(n - n_x{}^{(k)})(n - n_y{}^{(k)})}\right] = 3$$

Based on the numerical simulation, we also find that with the increase of n, the distribution of the normalized statistic $\hat{\rho}_{xy}{}^{(k)}$ of eqn. (S-2) will tend to standard normal distribution (Figure S2), and actually if $n > 100$, the statistic well follows normal distribution in general. Hence, we assume that if genes x and y are independent of each other, our normalized statistic follows standard normal distribution. We can use the normal distribution to test the independency or association of

any two genes in a cell k by $\hat{\rho}_{xy}{}^{(k)}$ of eqn. (S-2).

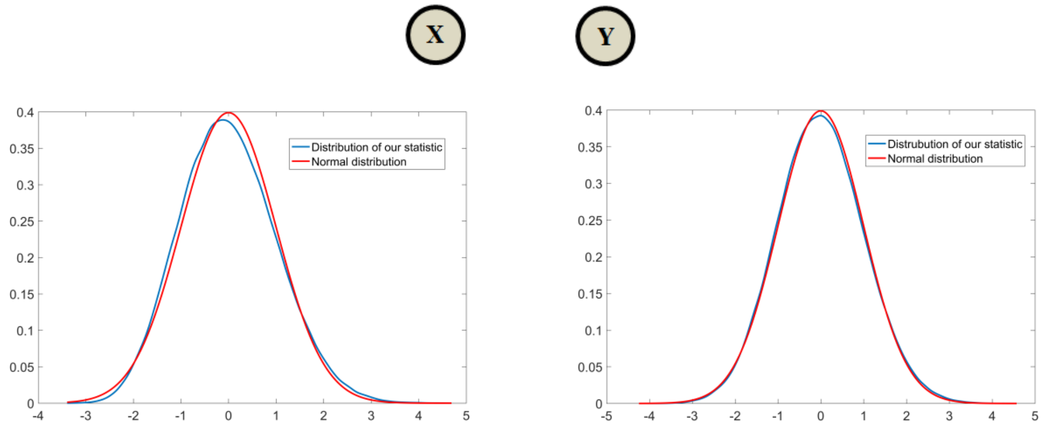

(A) n =100 **(B)** n = 500

Figure S2. The comparision of standard normal distribution and the distribution of $\hat{\rho}_{xy}{}^{(k)}$. The density function is calculated by kernel density estimation based on 200,000 plots, and n_x and n_y are equal to 0.2n. The two genes x and y are independent of each other.

(4) Performance of the statistic in different situations or associations

We have discussed the mean value, variance and distribution of the statistic $\hat{\rho}_{xy}{}^{(k)}$ when genes x and y are independent of each other. Figure S3 illustrates the performance of the normalized statistic when genes x and y follow different distributions. We can see no matter which distribution genes x and y follow, if only genes x and y are independent, the distribution of $\hat{\rho}_{xy}{}^{(k)}$ always approaches normal distribution and few plots are larger than the significant level.

Figure S4 illustrates the performance if genes x and y are correlated in partial cells and uncorrelated in the other cells. We can see no matter if the correlation is positive, negative or nonlinear, the distribution of our statistic always shows the double crest, which indicates that $\hat{\rho}_{xy}{}^{(k)}$ is able to distinguish the cells with the correlated genes and uncorrelated genes wonderfully.

Figure S5 illustrates the performance if genes x and y are correlated in all cells. We can see no matter if the dependency is linear, nonlinear or complex, the distribution of our statistic is always far from 0 and few plots are smaller than the significant level.

As a summary, our statistic model is able to distinguish the cells with the correlated genes and uncorrelated genes in a reliable manner.

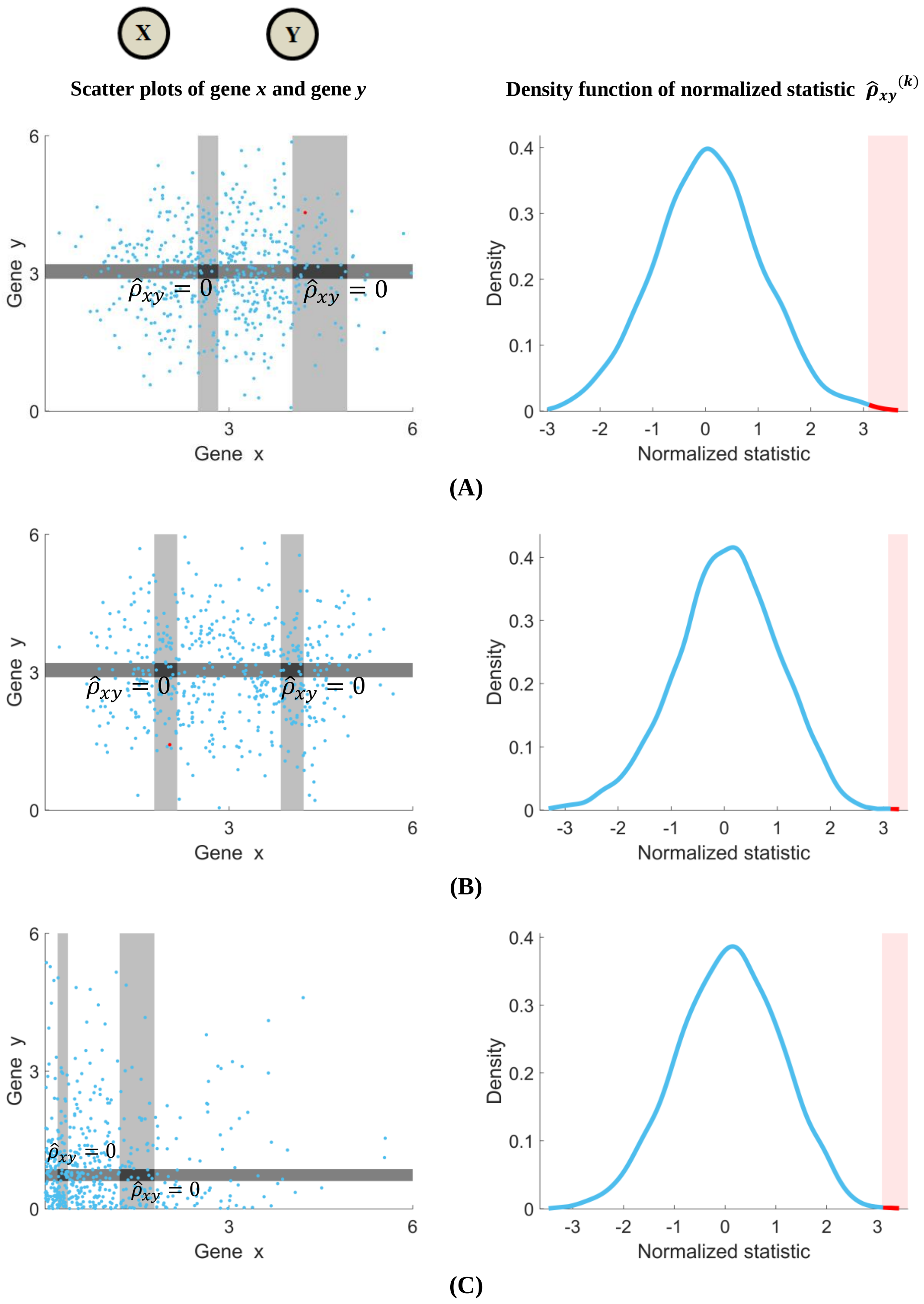

Figure S3. Performance of the statistic if genes *x* and *y* are independent of each other. $n_x{}^{(k)}$ and $n_y{}^{(k)}$ are set as 60 and n = 500. The box size will be changed with the density of plots, smaller in the dense area and larger in the sparse area. Thus, no matter which distribution the genes follow, the boxes cover the same number of plots, and the statistic model will not change. Red plots in the left figures represent $\hat{\rho}_{xy}{}^{(k)}$ in these plots are larger than the significant level of 0.001, which is corresponding with the red area in the right figures. We can see if genes *x* and *y* are independent, the distribution of our statistic is always similar to normal distribution and few plots are larger than the significant level.

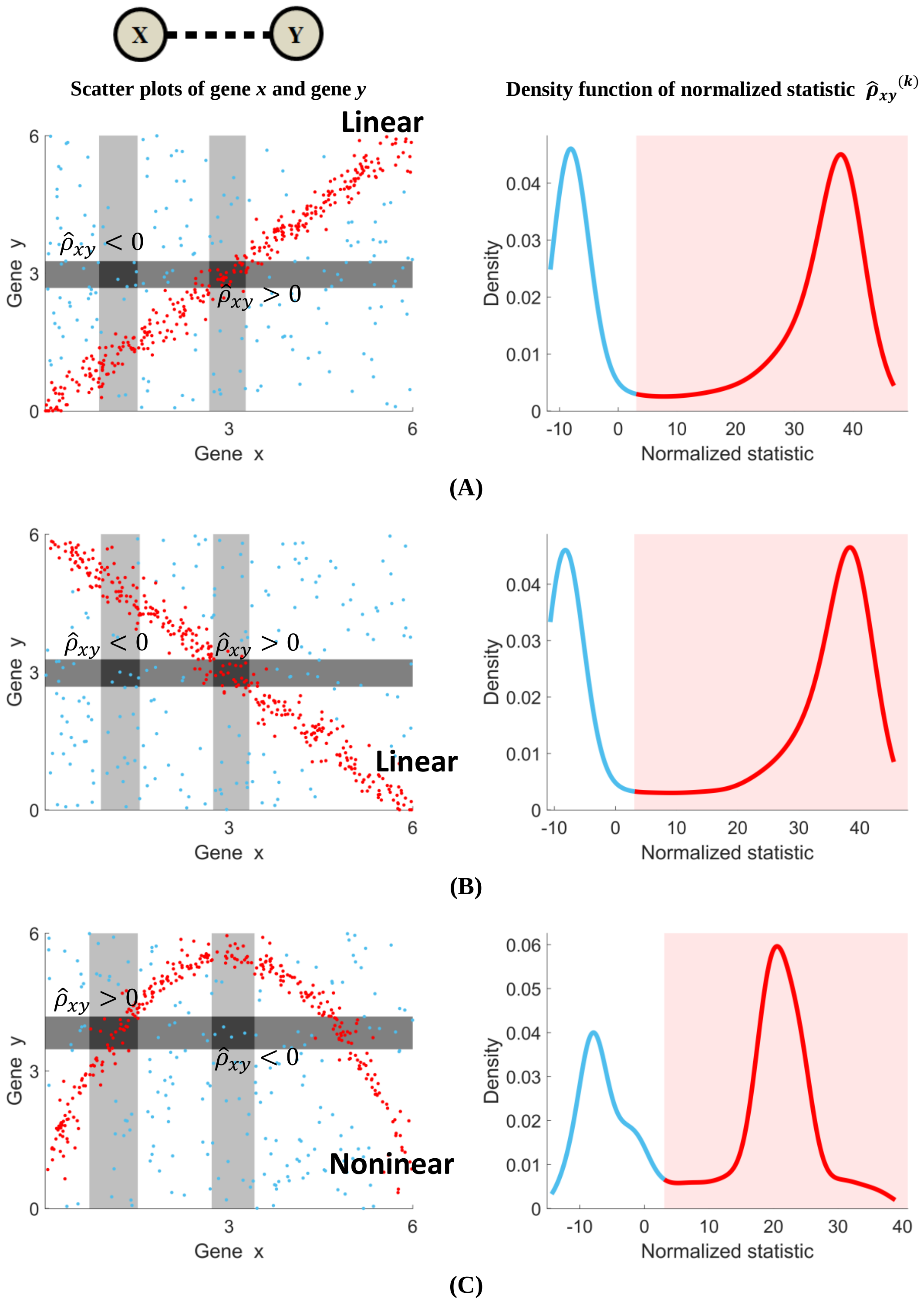

Figure S4. Performance of the statistic if genes x and y are correlated in partial cells and uncorrelated in the other cells. $n_x^{(k)}$ and $n_y^{(k)}$ are set as 60 and n = 500. Red plots in the left figures represent $\hat{\rho}_{xy}^{(k)}$ in these plots are larger than the significant level of 0.001, which is corresponding with the red area in the right figures. We can see the distribution of $\hat{\rho}_{xy}^{(k)}$ always shows the double crest and is able to distinguish the cells with the correlated genes and uncorrelated genes wonderfully, no matter if the correlation is positive, negative or nonlinear.

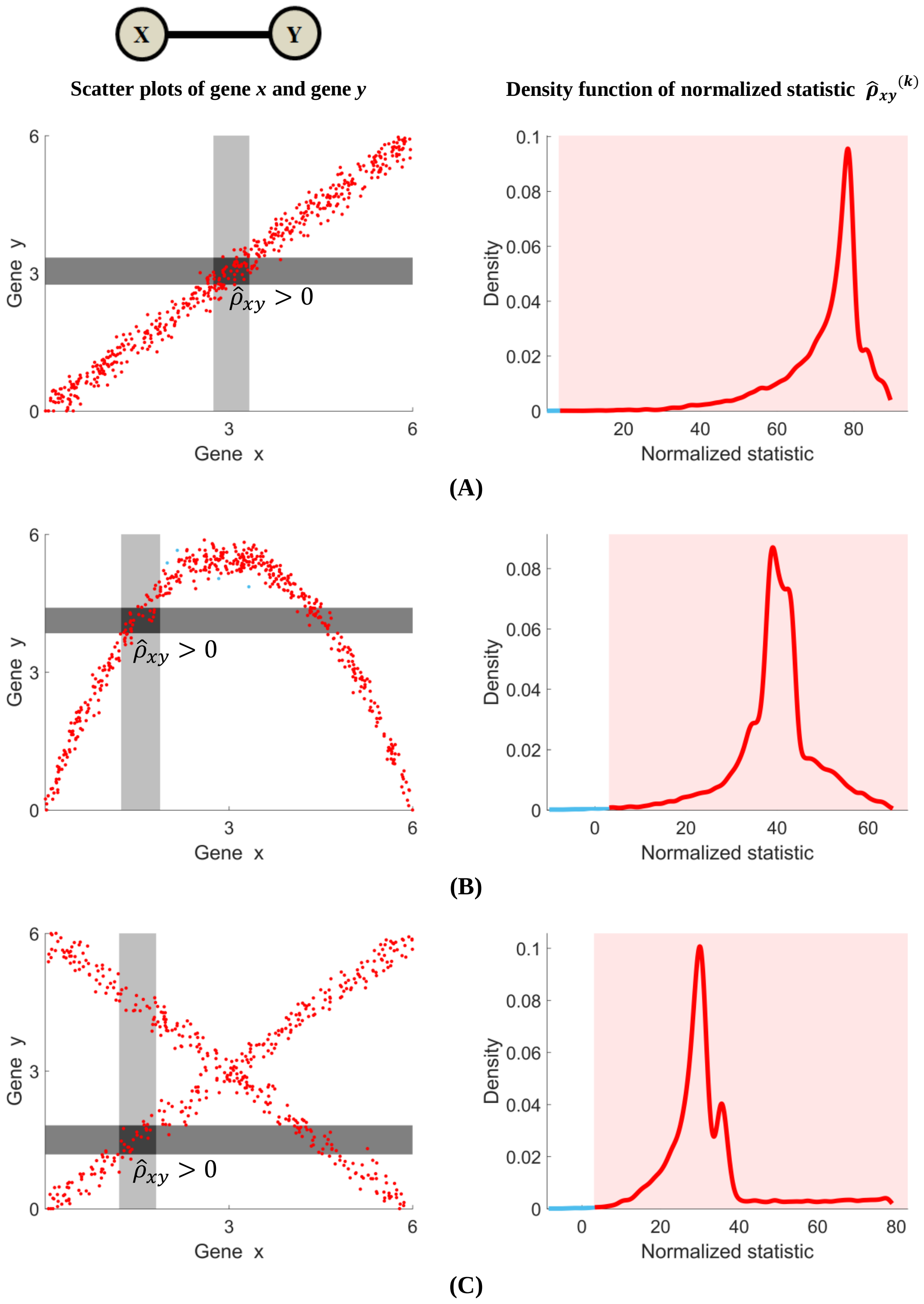

Figure S5. Performance of the statistic if genes x and y are correlated in all cells. $n_x^{(k)}$ and $n_y^{(k)}$ are set as 60 and n = 500. Red plots in the left figures represent $\hat{\rho}_{xy}^{(k)}$ in these plots are larger than the significant level of 0.001, which is corresponding with the red area in the right figures. We can see the correlation can be identified in most cells, no matter if the correlation is positive, negative or complex dependency.

(5) Performance of the statistic in the case of high dropout rate

Some experimental platforms such as Drop-seq and 10×genomics will produce the scRNA-seq data with high dropout rate, and then the gene expression matrix may be quite sparse. Figure S6 illustrates the performance of our statistic in the case of high dropout rate. If genes *x* and *y* are expressed in only 10% and 20% of total cells respectively and the expression level is 1, our statistic $\hat{\rho}_{xy}{}^{(k)}$ can only get four values: $\hat{\rho}_{11}$, $\hat{\rho}_{01}$, $\hat{\rho}_{10}$, $\hat{\rho}_{00}$. Then, as shown in Figure S6 (C, D), if genes *x* and *y* are independent, $\hat{\rho}_{xy}$ still follows normal distribution. If genes *x* and *y* are dependent, for example, Matthew Correlation Coefficient (MCC) = 0.33, $\hat{\rho}_{11}$ and $\hat{\rho}_{00}$ will be much larger than 0, and $\hat{\rho}_{01}$ and $\hat{\rho}_{10}$ will be much smaller than 0, which produce the double crest in the histogram. As a result, no matter in the case of high dropout rate or low dropout rate, our statistic gets similar performance, and thus we do not need to change our statistical model for the sparse gene expression matrix especially.

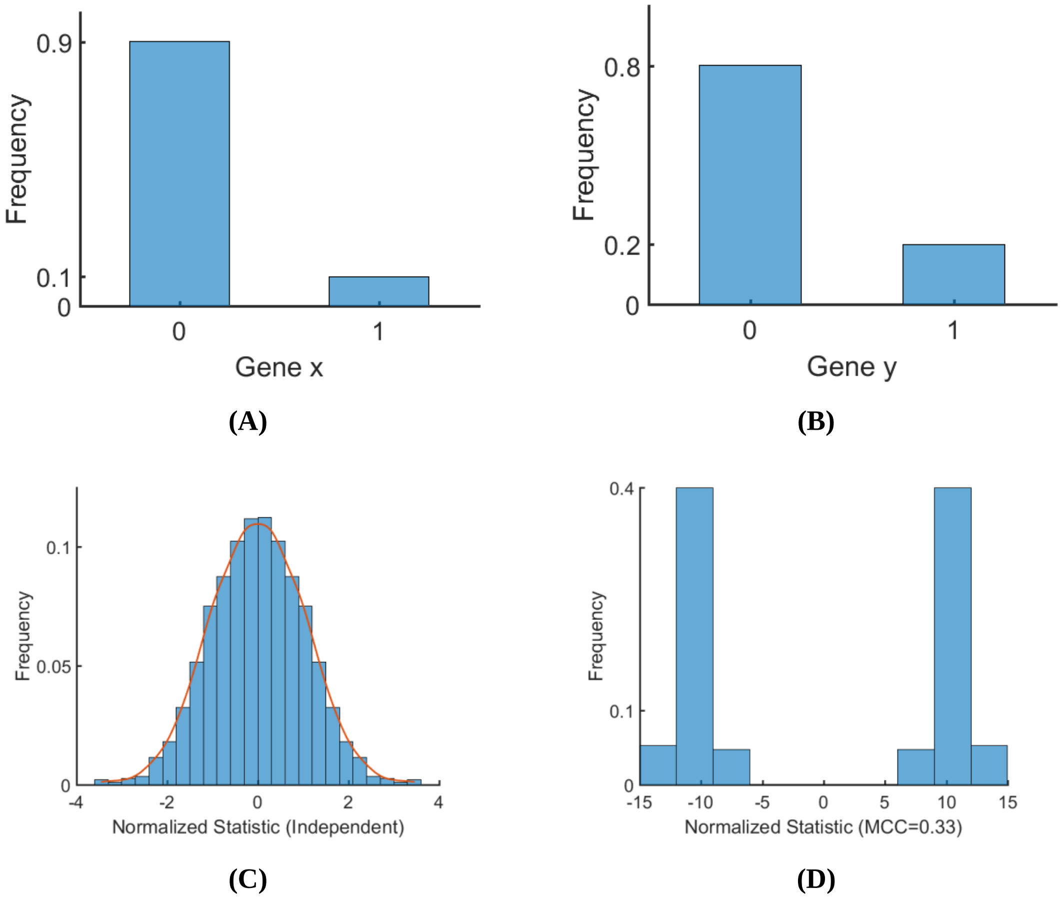

Figure S6. Performance of the statistic in the case of high dropout rate. **(A, B)** Histograms of the detected expression of genes *x* and *y*. **(C)** Histogram of the normalized statistic if genes *x* and *y* are independent. **(D)** Histogram of the normalized statistic if genes *x* and *y* are dependent (MCC = 0.33). Note that in (C) and (D), we made 1000 repetitions, and each figure was drawn from 4000 points (four points $\hat{\rho}_{11}$, $\hat{\rho}_{01}$, $\hat{\rho}_{10}$, $\hat{\rho}_{00}$ for each repetition).

Supplementary Note 2: The robustness of CSNs and NDM on different cell populations

If the dataset contains only one cell, it is impossible to construct the CSN; but if we have the expression profiles of more than one hundred cells, we can construct the network for each single cell, by exploring the distribution information from the population of cells. From the large number of cells in the dataset, we can estimate the joint distributions of every two genes, and these distributions can be used as the reference for CSN construction. However, if the composition of cells in the dataset is changed, for example, some new cells are added into the dataset, the reference or distribution might change with the new cells and then different CSNs would be constructed. In this section, we will discuss how CSNs change with the new cells or the composition of cells.

(1) First, if the proportion of each cell type in the new cells is the same as that in the original cells (i.e. the number of cells of each cell type increases with the same proportion), the distribution of each gene and the joint distribution $f(x, y)$ of each gene pair will not change, and then the statistical independency measurement $f(x, y) - f_X(x) f_Y(y)$ is fixed. As the statistic $\rho_{xy}^{(k)}$ in eqn. (S-1) is an approximation of $f(x_k, y_k) - f_X(x_k) f_Y(y_k)$, the expectation of the statistic is stable. Note that if we set $n_x^{(k)}$ and $n_y^{(k)}$ increase in proportion to n ($n_x^{(k)} = t_1 n$, $n_y^{(k)} = t_2 n$), the light grey, medium grey and dark grey boxes in Figure S1 ($D_x^{(k)}$, $D_y^{(k)}$ and $D_{xy}^{(k)}$ respectively) will barely change, and then

$$E\left(\rho_{xy}{}^{(k)}\right) = \frac{n_x{}^{(k)} n_y{}^{(k)}}{n^2}\left(\frac{p_{xy}}{p_x p_y} - 1\right) = t_1 t_2 \left(\frac{p_{xy}}{p_x p_y} - 1\right)$$

is a constant, where $p_x = \int_{D_x^{(k)}} f_X(x)dx$, $p_y = \int_{D_y^{(k)}} f_Y(y)dy$ and $p_{xy} = \iint_{D_{xy}^{(k)}} f(x,y)dxdy$ are the probability that a plot is located in the $D_x^{(k)}$, $D_y^{(k)}$ and $D_{xy}^{(k)}$ respectively. t_1 and t_2 are constants.

However, with the increase of n, the variance will be smaller, and then

$$E\left(\hat{\rho}_{xy}{}^{(k)}\right) = \frac{E\left(\frac{n_{xy}{}^{(k)}}{n} - \frac{n_x{}^{(k)} n_y{}^{(k)}}{n^2}\right)}{\sqrt{\frac{n_x{}^{(k)} n_y{}^{(k)} \left(n - n_x{}^{(k)}\right)\left(n - n_y{}^{(k)}\right)}{n^4 (n-1)}}} = \sqrt{n-1} \cdot \frac{t_1 t_2 \left(\frac{p_{xy}}{p_x p_y} - 1\right)}{\sqrt{t_1 t_2 (1 - t_1)(1 - t_2)}}$$

will be positive and become larger if $p_{xy} > p_x p_y$, negative and become smaller if $p_{xy} < p_x p_y$, and equal to 0 if $p_{xy} = p_x p_y$.

As a result, with the increase of n, the discrimination between the cells with the correlated genes and uncorrelated genes will become larger, the type II error will decrease, and some edges will be rediscovered in the new dataset.

(2) Next, if the new cells do not belong to any cell type in the original dataset (i.e. a new cell type is added into the dataset), the distribution of each gene and the joint distribution $f(x, y)$ of each gene pair will be changed, and then the CSNs constructed by the original dataset may be also changed. Here, to simplify the analysis, we suppose that there is the same distribution of each gene in the new cells as the original cells, but all of the genes do not have any correlations. Then, the new cells are obviously different from any cell type in the original dataset, and will interfere with the edge recognition in the CSN construction, which represents an extreme case.

We set $n_x^{(k)}$ and $n_y^{(k)}$ increase in proportion to sample size, number of the original cells is n_0, and number of the new cells is n. If CSNs are constructed by the original cells, then

$$E\left(\hat{\rho}_{xy}{}^{(k)}\right) = \sqrt{n_0 - 1} \cdot \frac{t_1 t_2 \left(\frac{p_{xy}}{p_x p_y} - 1\right)}{\sqrt{t_1 t_2 (1 - t_1)(1 - t_2)}}$$

On the other hand, if CSNs are constructed by the original cells and the new cells, then

$$E\left(\hat{\rho}_{xy}{}^{(k)\prime}\right) = \sqrt{n_0 + n - 1} \cdot \frac{t_1 t_2 \left(\frac{\frac{n_0 p_{xy} + n p_x p_y}{n_0 + n}}{p_x p_y} - 1\right)}{\sqrt{t_1 t_2 (1 - t_1)(1 - t_2)}}$$

Thus

$$\frac{E\left(\hat{\rho}_{xy}{}^{(k)\prime}\right)}{E\left(\hat{\rho}_{xy}{}^{(k)}\right)} = \frac{\sqrt{n_0 + n - 1} \cdot \frac{t_1 t_2 \left(\frac{n_0 p_{xy} + n p_x p_y}{p_x p_y (n_0 + n)} - 1\right)}{\sqrt{t_1 t_2 (1 - t_1)(1 - t_2)}}}{\sqrt{n_0 - 1} \cdot \frac{t_1 t_2 \left(\frac{p_{xy}}{p_x p_y} - 1\right)}{\sqrt{t_1 t_2 (1 - t_1)(1 - t_2)}}} = \frac{\sqrt{n_0 + n - 1} \cdot \frac{n_0}{n_0 + n}}{\sqrt{n_0 - 1}} \approx \frac{1}{\sqrt{1 + \frac{n}{n_0}}}$$

We can see the normalized statistic will become smaller with the increase of n. Compared with the CSNs constructed from the original dataset, the CSNs from the new dataset will lose some edges. However, if the significance of an edge (i.e. the normalized statistic) is sufficiently large, this edge can still be retained.

As a result, if a new cell type is added into the dataset, CSNs will lose some edges, but the significant edges are retained.

(3) Finally, if the new cells belong to partial cell types in the original dataset, the proportion of each cell type will change. It is obvious that to the cell types with more samples, more edges will be identified, while to the other cell types, the noise will increase and some edges will be lost. Hence, this situation is a combination of the two situations mentioned above.

As a conclusion, the robustness of an edge is determined by its normalized statistic; the larger normalized statistic, the more robust this edge is. In addition, if there are many or more cells in a cell type, the edges of the CSNs in this cell type can be identified more easily. Furthermore, the topological structure of CSN is stable and robust, which means the hub genes that have higher degrees in the CSN are always hub genes no matter how many edges can be identified to some extent.

On the other hand, we further transfer CSNs to a network degree matrix (NDM) to embody the network features and reduce the dimensions simultaneously. The value in this matrix is the number of edges connected to each gene in each CSN, which means that for gene x in the network of cell k

$$NDM_{xk} = \sum_{y=1, y \neq x}^{m} edge_{xy}{}^{(k)} \qquad \text{(S-3)}$$

Then we can get a matrix ***NDM*** with $m \times n$ elements. Moreover, in this work we normalize the ***NDM*** by

$$\widehat{NDM}_{xk} = \frac{NDM_{xk}}{\sum_{i=1}^{m} NDM_{ik}} \cdot \frac{a^2}{c} \tag{S-4}$$

where *a* is the average genes per cell in the scRNA-seq data and is defined as

$$a = \frac{1}{n}\sum_{j=1}^{n}\sum_{i=1}^{m} \mathrm{sgn}\left(GEM_{ij}\right)$$

c is a constant, and is chosen as 2000 in this work based on the computational simulation.

GEM is the original gene expression matrix and sgn() is sign function. Thus each cell has the same number of network degrees, which is determined by the average genes per cell in the scRNA-seq data. The normalization is able to improve the robustness and helps to the comparison of the cells from different cell populations. Hence, for NDM, we can easily conclude that if there are more cells in a cell type, there are more edges in the CSNs of this cell type, but if we normalize the NDM by eqn. (S-4), NDM will be robust.

Supplementary Note 3: The input, output and application fields of our CSN method

Input:

Gene expression matrix (RPKM/FPKM/TPM/count)

Significant level (e.g. 0.001, 0.01, 0.05 …). If the significant level is larger, it is easier to reject the null hypothesis and get more edges. In this paper, we set significant level as 0.01.

Box size. $n_x^{(k)}$ and $n_y^{(k)}$ should be determined in advance. In this paper, we set $n_x^{(k)}$ and $n_y^{(k)}$ approximately equal to $0.1n$. Users can change this parameter as well.

Output:

Cell-specific network for each cell (rows = genes, column = genes)

Network degree matrix (rows = genes, column = cells)

Normalized statistic of edge x-y for all cells ($1\times n$ vector)

Application fields:

The number of cells should be 100 at least.

Supplementary Note 4: Algorithms and their parameters used in clustering, visualization and pseudo-trajectory analysis

Preprocessing: GEM is preprocessed from initial gene expression matrix by normalization, gene selection (listed in Supplementary Note 5) and logarithm, i.e. $\hat{x} = \log(1 + x)$. NDM is straight transformed from GEM and also preprocessed by logarithm.

Hierarchical clustering

Distance between two objects: *Euclidean distance*

Algorithm for computing distance between clusters: *Ward's linkage* (Inner squared distance, minimum variance algorithm)

Number of clusters: *Same as the number of categories in the original dataset*

k-means clustering

Centroid initialization: *k-means++ algorithm* (1)

Distance between two objects: *Squared Euclidean distance*

Maximum number of iterations: *1000*

Number of times to repeat clustering using new initial cluster centroid positions: *100*

Number of clusters: *Same as the number of categories in the original dataset*

k-medoids clustering

Algorithm to find medoids: *Partitioning Around Medoids (PAM)* (2)

Centroid initialization: *k-means++ algorithm* (1)

Distance between two objects: *Squared Euclidean distance*

Maximum number of iterations: *1000*

Number of times to repeat clustering using new initial cluster centroid positions: *1*

Number of clusters: *Same as the number of categories in the original dataset*

SNN-Cliq (3)

Number of neighbors: *5*

Other parameters: *Default*

SIMLR (4)

Number of clusters: *Same as the number of categories in the original dataset*

Other parameters: *Default*

t-SNE (5)

In clustering analysis:

Preprocessed by PCA to reduce the dimensions to: *20*

t-SNE reduce the dimensions to: *5*

Perplexity: *30*

Clustering method: *Hierarchical clustering / k-means*

In visualization:

Preprocessed by PCA to reduce the dimensions to: *20*

t-SNE reduce the dimensions to: *2*
Perplexity: *30*

Wanderlust (6)
Preprocessed by PCA to reduce the dimensions to: *250 (Chu-time) / 200 (Trapnell)*
Branch: *no branch*
Other parameters: *Default*

Supplementary Note 5: Datasets used for validation of CSN

Ten single-cell RNA-seq datasets (7-15) and one bulk RNA-seq dataset are used in this paper. The normalization, gene selection rules and data sources of all datasets are listed below.

Dataset	Number of cells	Number of cell types	Normalization method	Number of genes used in GEM and NDM	Gene selection rules	Data Sources
Buettner	182	3	FPKM	8988	FPKM per cell on average > 5	*Supplementary Data 2*
Kolodziejczyk	704	3	count	10685	Counts per cell on average > 10	ArrayExpress (http://www.ebi.ac.uk/arrayexpress) *E-MTAB-2600*
Pollen	249	11	TPM	14805	Expressed in at least 10 cells	NCBI Sequence Read Archive (http://www.ncbi.nlm.nih.gov/Traces/sra/) *SRP041736*
Zeisel	3005	9	TPM	15964		Gene Expression Omnibus (www.ncbi.nlm.nih.gov/geo) *GSE60361*
Darmanis	420	8	TPM	15651		*GSE67835*
Chu-type	1018	7	FPKM	16619		*GSE75748*
Chu-time	758	6	FPKM	15691		*GSE75748*
Trapnell	372	4	FPKM	17867		*GSE52529*
Kim	118	3	TPM	13392		*GSE73121*
Xin	1600	4	RPKM	25040		*GSE81608*
TCGA	1135	4	FPKM	33409	Expressed in at least 500 samples	The Cancer Genome Atlas (TCGA) *Project: TCGA-LUAD and TCGA-LUSC* (https://cancergenome.nih.gov)

Supplementary Note 6: The relevance between correlation coefficient and normalized statistic

We selected 500 genes with the largest variance on *Chu-type* dataset, and then calculated the squared correlation coefficient R^2 and average normalized statistic for every two genes in H1 cells. Figure S7 indicates the high relevance between R^2 and the normalized statistic of eqn. (S-2). As shown in the figure, a few points get the large R^2 but normalized statistic is almost zero, which is because the two genes in most cells are not expressed except one or two cells, and thus R^2 approaches 1 by only those cells.

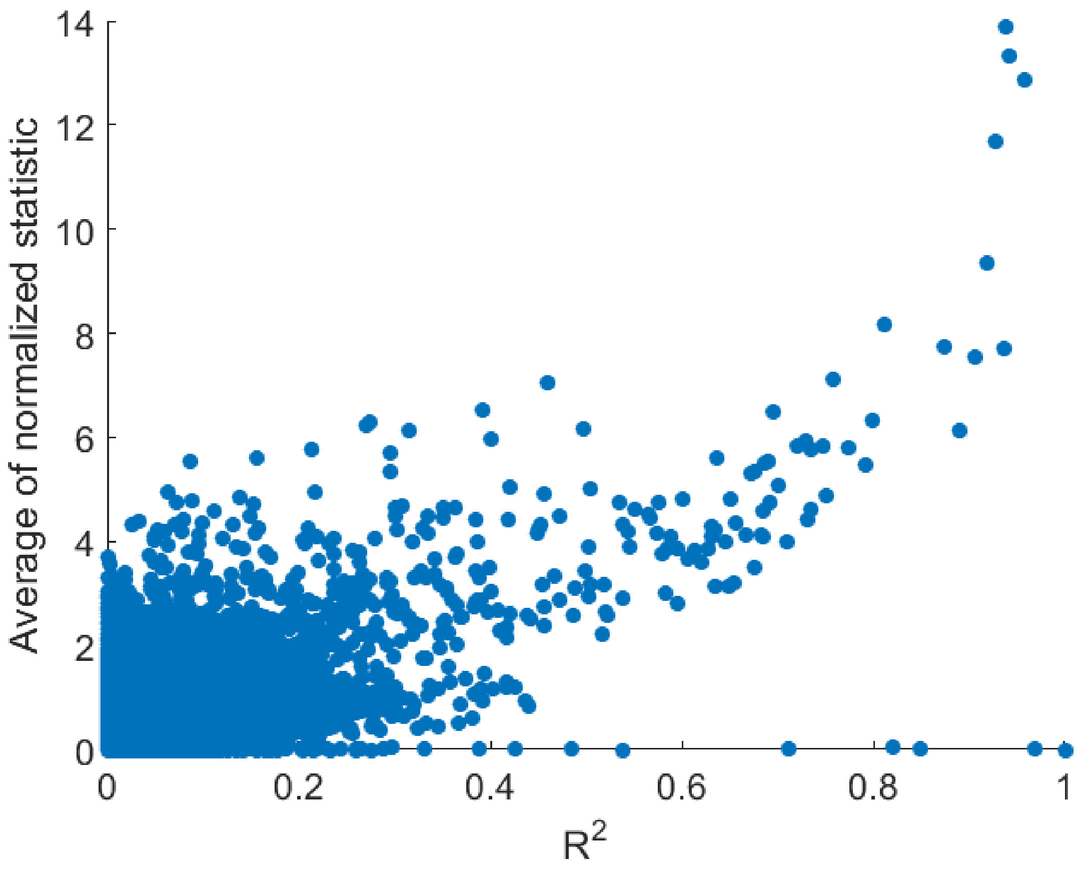

Figure S7. The relevance between squared correlation coefficient and average normalized statistic in H1 cells.

Supplementary Note 7: Illustration of some gene associations

Figure S8 illustrates some gene associations with POU5F1 based on *Chu-type* dataset. Some literatures have validated the gene interactions between POU5F1 and CDH1 (16), POU5F1 and EPAS1 (17), POU5F1 and NANOG (18), which indicates that it is possible to find the information of gene associations by scRNA-seq data.

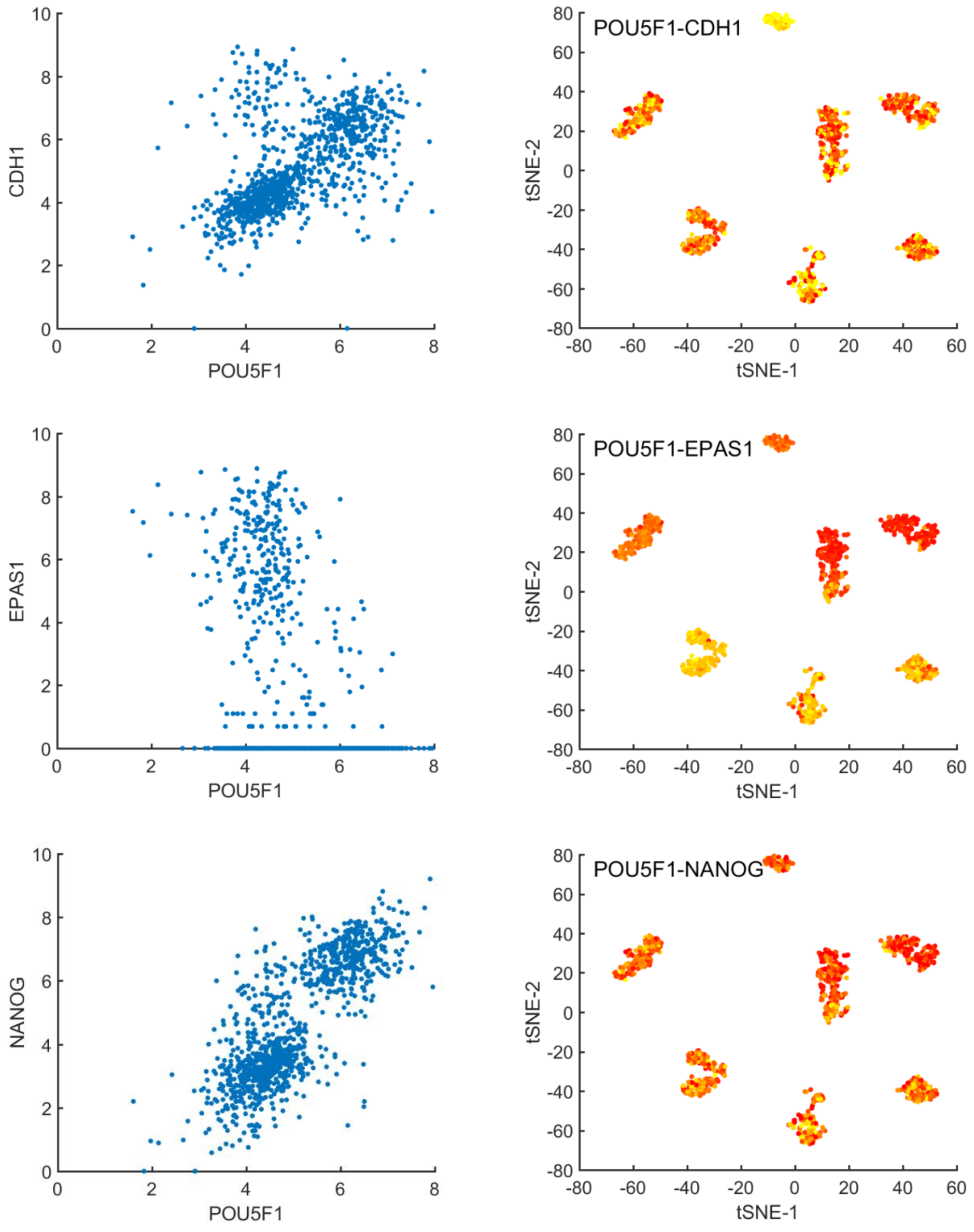

Figure S8. Scatter diagrams and performance of the normalized statistic of edge POU5F1 - CDH1, POU5F1 - EPAS1 and POU5F1 – NANOG.

Supplementary Note 8: The top genes with the highest degrees on Chu-type dataset

We listed the top 10 genes with the highest degrees of each cell type on *Chu-type* dataset.

H1	H9	DEC	EC	HFF	NPC	TB
PHC1	DPPA4	CER1	PECAM1	LIN28A	CDH6	ITGB6
L1TD1	L1TD1	SERPINB9	CXorf36	B2M	LIX1	VGLL1
TERF1	PHC1	POU2AF1	MMRN2	AXL	LRP2	PLSCR5
DPPA4	TERF1	ERBB4	TFPI	LGALS1	PAX6	GABRP
TDGF1	POU5F1	GATA4	F2RL2	ANXA2	ILDR2	PIK3C2G
POLR3G	ZFP42	RHOBTB3	TNFSF10	CD24	DLK1	PAPPA2
USP44	HSPD1	STC1	SPTBN1	CD59	DPYSL5	GUCY1A3
DNMT3B	USP44	CST1	SCARF1	THBS1	OTX1	HAND1
POU5F1	CD24	FGF17	CARD8	COL1A1	TMSB4X	TPM1
S100A11	SNURF	MYCT1	SOX7	TWSG1	EFNA5	P2RY6

Supplementary Note 9: The "darker" genes of each cell type on Chu-type dataset

We listed the "darker" genes of each cell type on *Chu-type* dataset (p-value in this table is the maximum p-value among the top-expressed cell type and other cell type, and Wilcoxon rank-sum test is used).

Gene	Cell type	p-value of network degree	p-value of expression	Gene	Cell type	p-value of network degree	p-value of expression
TDGF1	H1	3.59E-06	0.956	MSL1	EC	3.32E-06	0.010
VSNL1	H1	3.62E-08	0.094	PHF8	EC	1.07E-06	0.086
DNAH11	H9	2.00E-06	0.030	GORAB	EC	1.01E-06	0.573
NPPB	HFF	1.66E-07	0.015	KANSL3	EC	1.51E-07	0.026
TMEM97	NPC	3.33E-06	0.053	EFNA2	EC	1.01E-07	0.017
SGPL1	NPC	7.88E-06	0.818	UBQLN4	EC	2.14E-06	0.061
ICAM1	NPC	2.18E-07	0.038	SIK2	EC	3.12E-06	0.016
ARSA	NPC	6.86E-06	0.019	FBXO33	EC	6.57E-06	0.794
MX2	DEC	9.35E-06	0.015	KANSL1L	EC	1.01E-06	0.016
GATA2	DEC	4.92E-06	0.504	STAT5B	EC	3.74E-07	0.031
ACTA2	DEC	3.39E-12	0.022	ASB3	EC	6.23E-07	0.024
LGALS3	DEC	1.73E-16	0.337	THAP3	EC	1.60E-06	0.017
TNFRSF19	DEC	5.34E-06	0.016	TTC32	EC	8.76E-06	0.195
FAM131B	EC	6.30E-06	0.012	ZHX2	EC	2.03E-06	0.053
PHF13	EC	2.96E-06	0.026	TAF1C	EC	5.64E-06	0.151
ZNF226	EC	1.39E-06	0.904	ZNF618	EC	2.45E-07	0.095
IFT122	EC	3.08E-06	0.014	C1orf21	EC	2.49E-06	0.024
CDH8	EC	4.98E-06	0.012	AGBL5	EC	1.75E-07	0.042
EZH1	EC	2.60E-08	0.028	EXOC6	EC	6.33E-06	0.014
KHDRBS3	EC	2.09E-07	0.033	SCMH1	EC	1.67E-07	0.188
IDH2	EC	2.26E-07	0.051	DOCK3	EC	1.43E-06	0.044
EFCAB7	EC	4.92E-08	0.024	FXR2	TB	9.83E-06	0.017

Supplementary Note 10: Results of clustering analysis

We used different measurements to evaluate the performances of clustering analysis, which are shown below.

(1) ***Adjusted Random Index (ARI)***

		Buettner	Kolod ziejczyk	Pollen	Zeisel	Darmanis	Chu-type	Chu-time	Kim	Trapnell
Hierarchical	GEM	0.48	0.49	0.95	0.55	0.63	0.75	0.67	0.66	0.08
	**NDM**	0.82	0.99	0.96	0.53	0.91	0.77	0.72	0.73	0.24
k-means	GEM	0.31	0.53	0.90	0.39	0.58	0.73	0.59	0.60	0.14
	**NDM**	0.74	0.80	0.87	0.43	0.77	0.77	0.70	0.83	0.44
Hierarchical (tSNE)	GEM	0.32	0.99	0.94	0.60	0.67	0.98	0.68	0.66	0.16
	**NDM**	0.97	1.00	0.85	0.62	0.86	0.99	0.68	1.00	0.43
k-means (tSNE)	GEM	0.30	0.99	0.94	0.62	0.65	0.98	0.69	0.72	0.16
	**NDM**	0.94	1.00	0.85	0.65	0.85	0.99	0.69	1.00	0.47
k-medoids	GEM	0.14	0.03	0.91	0.43	0.36	0.60	0.43	0.57	0.00
	**NDM**	0.31	0.73	0.89	0.11	0.23	0.76	0.41	0.61	0.23
SIMLR	GEM	0.92	0.99	0.90	0.56	0.75	0.74	0.66	0.97	0.21
	**NDM**	1.00	1.00	0.92	0.67	0.90	0.75	0.67	0.95	0.31
SNN-Cliq	GEM	0.00	0.00	0.90	0.50	0.20	0.64	0.30	0.58	0.00
	**NDM**	0.50	0.65	0.90	0.60	0.01	0.61	0.36	0.58	0.24

(2) ***F1-measure***

		Buettner	Kolod ziejczyk	Pollen	Zeisel	Darmanis	Chu-type	Chu-time	Kim	Trapnell
Hierarchical	GEM	0.66	0.67	0.95	0.63	0.69	0.79	0.73	0.78	0.35
	**NDM**	0.88	0.99	0.96	0.61	0.93	0.81	0.77	0.82	0.49
k-means	GEM	0.54	0.70	0.91	0.48	0.65	0.78	0.67	0.73	0.36
	**NDM**	0.83	0.87	0.88	0.54	0.82	0.81	0.76	0.89	0.60
Hierarchical (tSNE)	GEM	0.55	0.99	0.95	0.67	0.73	0.98	0.74	0.78	0.38
	**NDM**	0.98	1.00	0.86	0.69	0.88	0.99	0.74	1.00	0.60
k-means (tSNE)	GEM	0.54	0.99	0.95	0.68	0.71	0.98	0.75	0.82	0.38
	**NDM**	0.96	1.00	0.86	0.71	0.88	0.99	0.75	1.00	0.62
k-medoids	GEM	0.46	0.50	0.92	0.52	0.48	0.67	0.54	0.72	0.37
	**NDM**	0.55	0.82	0.91	0.34	0.40	0.79	0.52	0.75	0.49
SIMLR	GEM	0.95	0.99	0.91	0.63	0.80	0.78	0.73	0.98	0.41
	**NDM**	1.00	1.00	0.92	0.73	0.92	0.79	0.74	0.97	0.51
SNN-Cliq	GEM	0.50	0.52	0.91	0.58	0.39	0.70	0.48	0.72	0.37
	**NDM**	0.69	0.75	0.91	0.67	0.34	0.67	0.47	0.74	0.48

(3) *Purity*

		Buttner	Kolod ziejczyk	Pollen	Zeisel	Darmanis	Chu-type	Chu-time	Kim	Trapnell
Hierarchical	GEM	0.77	0.75	0.97	0.80	0.85	0.83	0.80	0.84	0.42
	**NDM**	0.93	1.00	0.97	0.77	0.94	0.86	0.87	0.89	0.48
k-means	GEM	0.70	0.76	0.92	0.78	0.84	0.82	0.75	0.81	0.45
	**NDM**	0.91	0.92	0.89	0.74	0.83	0.87	0.87	0.93	0.65
Hierarchical (tSNE)	GEM	0.67	1.00	0.96	0.89	0.89	0.99	0.83	0.84	0.46
	**NDM**	0.99	1.00	0.91	0.88	0.94	1.00	0.82	1.00	0.67
k-means (tSNE)	GEM	0.68	1.00	0.96	0.89	0.89	0.99	0.83	0.88	0.46
	**NDM**	0.98	1.00	0.91	0.88	0.94	1.00	0.83	1.00	0.67
k-medoids	GEM	0.56	0.50	0.93	0.73	0.71	0.75	0.67	0.82	0.30
	**NDM**	0.65	0.89	0.92	0.46	0.50	0.87	0.68	0.77	0.50
SIMLR	GEM	0.97	1.00	0.94	0.86	0.90	0.84	0.82	0.99	0.48
	**NDM**	1.00	1.00	0.95	0.91	0.93	0.84	0.79	0.98	0.56
SNN-Cliq	GEM	0.36	0.42	0.94	0.83	0.68	0.77	0.54	0.81	0.29
	**NDM**	0.73	0.94	0.91	0.87	0.34	0.89	0.70	0.76	0.55

(4) *Entropy*

		Buettner	Kolod ziejczyk	Pollen	Zeisel	Darmanis	Chu-type	Chu-time	Kim	Trapnell
Hierarchical	GEM	0.77	0.72	0.12	0.78	0.53	0.43	0.62	0.54	1.76
	**NDM**	0.31	0.02	0.10	0.86	0.29	0.32	0.52	0.45	1.30
k-means	GEM	1.02	0.69	0.28	0.93	0.64	0.46	0.77	0.66	1.66
	**NDM**	0.49	0.31	0.34	1.06	0.66	0.32	0.54	0.32	1.01
Hierarchical (tSNE)	GEM	1.02	0.01	0.14	0.52	0.30	0.07	0.55	0.52	1.62
	**NDM**	0.07	0.00	0.28	0.59	0.21	0.03	0.55	0.00	1.02
k-means (tSNE)	GEM	1.02	0.01	0.14	0.51	0.31	0.07	0.53	0.46	1.62
	**NDM**	0.12	0.00	0.28	0.57	0.23	0.03	0.52	0.00	0.98
k-medoids	GEM	1.19	1.42	0.29	1.10	1.13	0.71	1.10	0.69	1.95
	**NDM**	1.03	0.45	0.27	2.12	1.61	0.37	1.13	0.63	1.30
SIMLR	GEM	0.14	0.01	0.24	0.64	0.36	0.43	0.57	0.06	1.50
	**NDM**	0.00	0.00	0.16	0.50	0.28	0.36	0.62	0.11	1.20
SNN-Cliq	GEM	1.58	1.54	0.27	0.69	1.39	0.66	1.72	0.68	1.98
	**NDM**	0.80	0.26	0.21	0.65	2.49	0.28	1.04	0.62	1.14

Supplementary Note 11: Results of visualization

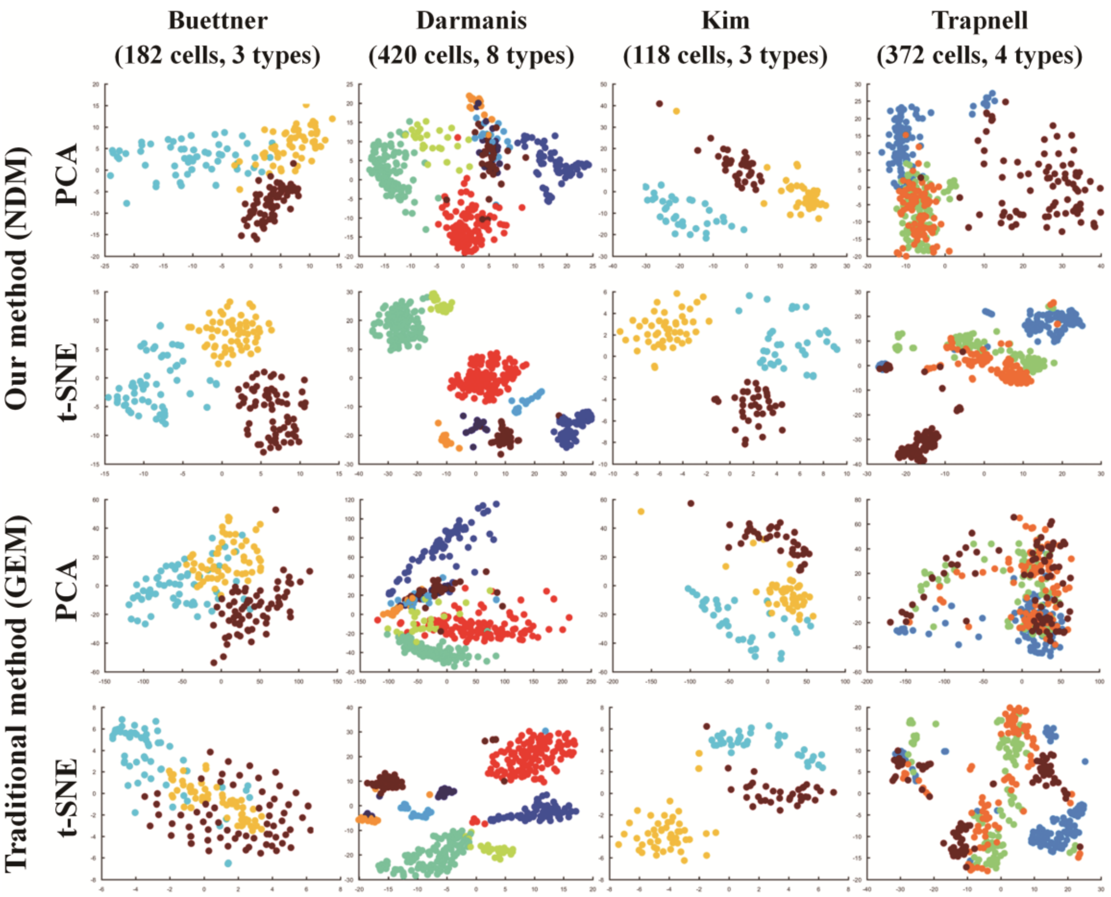

Figure S9. The clustering performances of NDM and GEM on four datasets. PCA and t-SNE that represent linear and nonlinear dimension-reduction method respectively are used for visualization. Different colors represent different cell types. x-axis and y-axis in each graph represent PC1 and PC2 (or tSNE1 and tSNE2), respectively.

Supplementary Note 12: Results of pseudo trajectory analysis

In this paper, we used two datasets with the gold standard from literatures, which include 758 cells with 6 stages (0h, 12h, 24h, 36h, 72h, 96h) in Chu-time dataset (14) and 372 cells with 4 stages (0h, 24h, 48h, 72h) in Trapnell dataset (10). Wanderlust (6) is a method to construct no-branch pseudo trajectory, which gives each cell a value to represent the cell order, and the cells in the later stages will get larger values. GEM and NDM are used for comparison. Figure S10 illustrates that the Wanderlust values increase in accordance with the time sequence in Chu-time dataset, and the results of GEM and NDM are quite similar. But in Trapnell dataset, NDM is able to identify the change at 72h, but GEM fails.

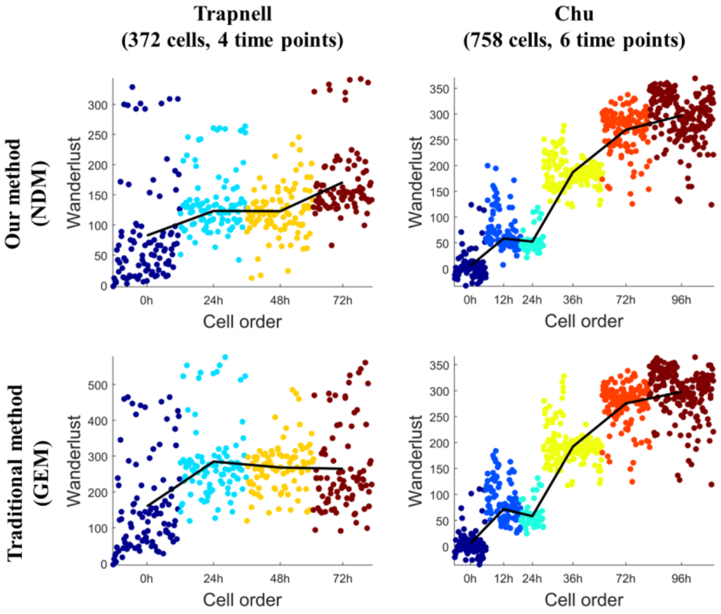

Figure S10. The comparison of GEM and NDM in pseudo trajectory analysis. The cells in the later stages will get larger Wanderlust values, and the average at every time points is shown as black line.

Supplementary Note 13: Comparison of different parameters of CSN

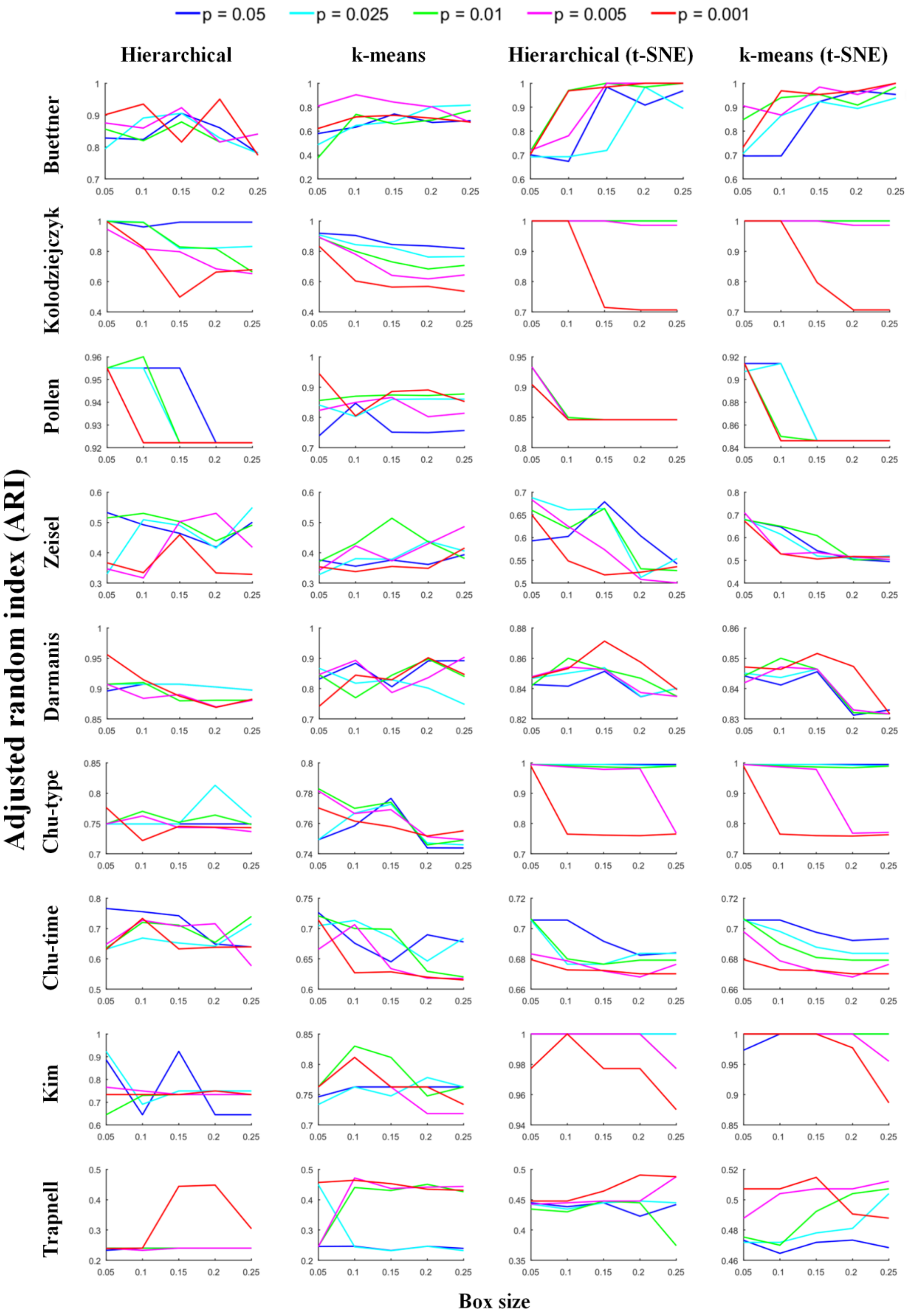

Figure S11. Comparison of different parameters of CSN. X-axis is box size and different colors represent different *p*-value. Y-axis is the ARI in clustering analysis. The results show that the optimum box size is about 0.1, and the optimum p-value is about 0.01, on average.

Supplementary Note 14: Comparison of different normalization methods

ARI of GEM and NDM in clustering analysis between different normalization methods is listed below. We can see NDM from the GEM normalized by TPM/FPKM/count gets the similar performances on the same dataset, though the result by TPM seems to be better. Thus, our NDM method is not sensitive to the normalization method, and is suitable to various types of gene expression matrix.

	Kolodziejczyk (NDM)		Kolodziejczyk (GEM)		Pollen (NDM)		Pollen (GEM)	
	TPM	count	TPM	count	TPM	count	TPM	Count
Hierarchical	1	0.99	0.73	0.49	0.96	0.92	0.95	0.95
k-means	0.93	0.80	0.70	0.53	0.87	0.82	0.90	0.87
Hierarchical (tSNE)	1	1	0.73	0.99	0.85	0.85	0.94	0.88
k-means (tSNE)	1	1	0.73	0.99	0.85	0.85	0.94	0.92

	Zeisel (NDM)		Zeisel (GEM)		Darmanis (NDM)		Darmanis (GEM)	
	TPM	count	TPM	count	TPM	count	TPM	Count
Hierarchical	0.53	0.56	0.55	0.39	0.91	0.89	0.63	0.46
k-means	0.43	0.41	0.39	0.36	0.77	0.80	0.58	0.42
Hierarchical (tSNE)	0.62	0.59	0.60	0.58	0.86	0.85	0.67	0.62
k-means (tSNE)	0.65	0.55	0.62	0.57	0.85	0.85	0.65	0.61

	Chu-type (NDM)		Chu-type (GEM)		Chu-time (NDM)		Chu-time (GEM)	
	TPM	FPKM	TPM	FPKM	TPM	FPKM	TPM	FPKM
Hierarchical	0.75	0.77	0.75	0.75	0.67	0.72	0.79	0.67
k-means	0.75	0.77	0.75	0.73	0.72	0.70	0.72	0.59
Hierarchical (tSNE)	0.99	0.99	0.99	0.98	0.72	0.68	0.72	0.68
k-means (tSNE)	0.99	0.99	0.99	0.98	0.72	0.69	0.72	0.69

	Kim (NDM)		Kim (GEM)		Trapnell (NDM)		Trapnell (GEM)	
	TPM	FPKM	TPM	FPKM	TPM	FPKM	TPM	FPKM
Hierarchical	0.73	0.73	0.66	0.66	0.24	0.24	0.08	0.08
k-means	0.83	0.75	0.60	0.56	0.48	0.44	0.13	0.14
Hierarchical (tSNE)	1	1	0.66	0.95	0.44	0.43	0.20	0.16
k-means (tSNE)	1	1	0.72	0.76	0.5	0.47	0.20	0.16

Supplementary Note 15: Comparison of different gene selection rules

ARI of GEM and NDM in clustering analysis between different gene selection rules is listed below. We can see that the different gene selection rules have just a little influence on the performance of GEM and NDM, and NDM is still superior to GEM clearly.

	Buettner (NDM)				Buettner (GEM)			
	FPKM per cell on average				FPKM per cell on average			
	> 1	> 5	> 10	> 50	> 1	> 5	> 10	> 50
Hierarchical	0.91	0.82	0.85	0.85	0.26	0.48	0.34	0.38
k-means	0.90	0.74	0.85	0.75	0.30	0.31	0.25	0.31
Hierarchical (tSNE)	0.78	0.97	0.70	0.95	0.37	0.32	0.29	0.38
k-means (tSNE)	0.84	0.94	0.71	0.95	0.30	0.30	0.31	0.42

	Darmanis (NDM)				Darmanis (GEM)			
	Detected expression in at least				Detected expression in at least			
	1 cell	5 cells	10 cells	50 cells	1 cell	5 cells	10 cells	50 cells
Hierarchical	0.88	0.91	0.91	0.90	0.63	0.64	0.63	0.59
k-means	0.76	0.90	0.77	0.90	0.58	0.57	0.58	0.57
Hierarchical (tSNE)	0.85	0.85	0.86	0.64	0.64	0.67	0.67	0.63
k-means (tSNE)	0.85	0.85	0.85	0.64	0.65	0.65	0.65	0.63

	Chu-type (NDM)				Chu-type (GEM)			
	Detected expression in at least				Detected expression in at least			
	1 cell	5 cells	10 cells	50 cells	1 cell	5 cells	10 cells	50 cells
Hierarchical	0.75	0.75	0.77	0.77	0.75	0.75	0.75	0.75
k-means	0.77	0.77	0.77	0.77	0.74	0.73	0.73	0.74
Hierarchical (tSNE)	0.99	0.99	0.99	0.99	0.98	0.98	0.98	0.98
k-means (tSNE)	0.99	0.99	0.99	0.99	0.98	0.98	0.98	0.98

	Chu-time (NDM)				Chu-time (GEM)			
	Detected expression in at least				Detected expression in at least			
	1 cell	5 cells	10 cells	50 cells	1 cell	5 cells	10 cells	50 cells
Hierarchical	0.64	0.65	0.72	0.75	0.67	0.68	0.67	0.67
k-means	0.70	0.70	0.70	0.71	0.60	0.59	0.59	0.59
Hierarchical (tSNE)	0.68	0.68	0.68	0.68	0.68	0.68	0.68	0.68
k-means (tSNE)	0.69	0.68	0.69	0.68	0.69	0.68	0.69	0.69

	Trapnell (NDM)				Trapnell (GEM)			
	Detected expression in at least				Detected expression in at least			
	1 cell	5 cells	10 cells	50 cells	1 cell	5 cells	10 cells	50 cells
Hierarchical	0.23	0.24	0.24	0.23	0.06	0.06	0.08	0.01
k-means	0.25	0.25	0.44	0.23	0.13	0.15	0.16	0.13
Hierarchical (tSNE)	0.43	0.45	0.43	0.44	0.17	0.16	0.16	0.16
k-means (tSNE)	0.47	0.50	0.47	0.47	0.16	0.16	0.16	0.16

	Kim (NDM)				Kim (GEM)			
	Detected expression in at least				Detected expression in at least			
	1 cell	5 cells	10 cells	50 cells	1 cell	5 cells	10 cells	50 cells
Hierarchical	0.77	0.73	0.73	0.72	0.66	0.66	0.66	0.73
k-means	0.78	0.76	0.83	0.73	0.61	0.61	0.60	0.56
Hierarchical (tSNE)	1.00	1.00	1.00	1.00	0.66	0.66	0.66	0.76
k-means (tSNE)	1.00	1.00	1.00	0.98	0.72	0.72	0.72	0.75

	Kolodziejczyk (NDM)			Kolodziejczyk (GEM)		
	Counts per cell on average			Counts per cell on average		
	> 10	> 20	> 50	> 10	> 20	> 50
Hierarchical	0.99	0.81	0.83	0.49	0.51	0.53
k-means	0.80	0.76	0.61	0.53	0.53	0.52
Hierarchical (tSNE)	1.00	1.00	1.00	0.99	0.99	0.99
k-means (tSNE)	1.00	1.00	1.00	0.99	0.99	0.99

	Pollen (NDM)			Pollen (GEM)		
	Detected expression in at least			Detected expression in at least		
	10 cells	20 cells	50 cells	10 cell	20 cells	50 cells
Hierarchical	0.96	0.96	0.92	0.95	0.94	0.95
k-means	0.87	0.84	0.90	0.90	0.91	0.92
Hierarchical (tSNE)	0.85	0.85	0.88	0.94	0.94	0.95
k-means (tSNE)	0.85	0.86	0.88	0.94	0.94	0.94

Supplementary Note 16: Clustering analysis for the imputed data

In this work, we used scImpute (18) to impute the dataset, and then constructed the NDM from the imputed data. Clustering methods including hierarchical clustering algorithm (HCA) and k-means were used for comparison. We also performed the clustering to the data that are preprocessed by t-SNE.

The result is shown in Figure S12. From Figure S12, we can see that the imputed GEM gets better results than original GEM in some datasets, but is usually inferior to the performance of NDM from original GEM. The result of NDM from imputed GEM is a little better than imputed GEM, but is obviously worse than NDM from original GEM. As a conclusion, the imputation based on the current methods is not recommended before CSN construction. In other words, existing imputation method is based on the expression level of scRNA-seq data but does not take into account of the gene-gene interactions, and thus, the identification of edges in CSNs from imputed data may be interfered, which leads to the worse result for the NDM from imputed data.

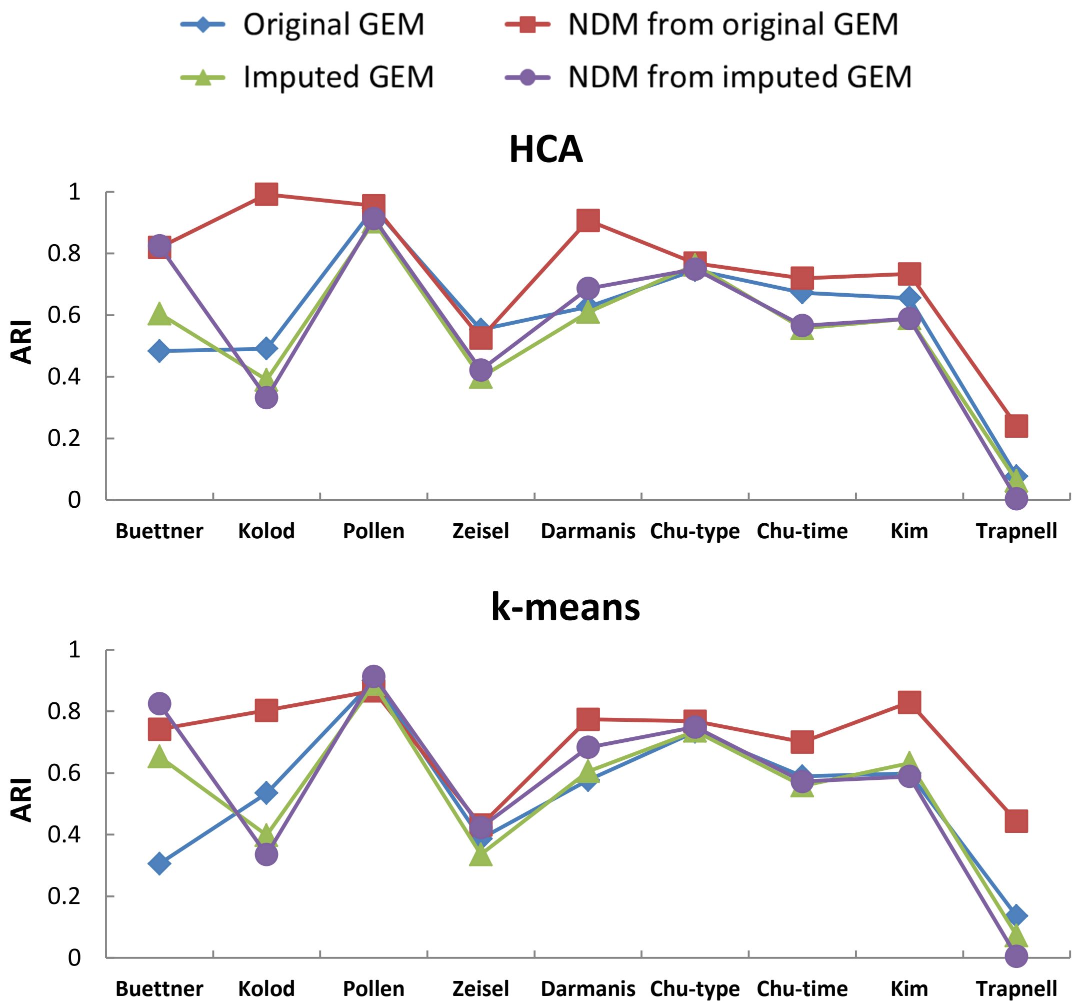

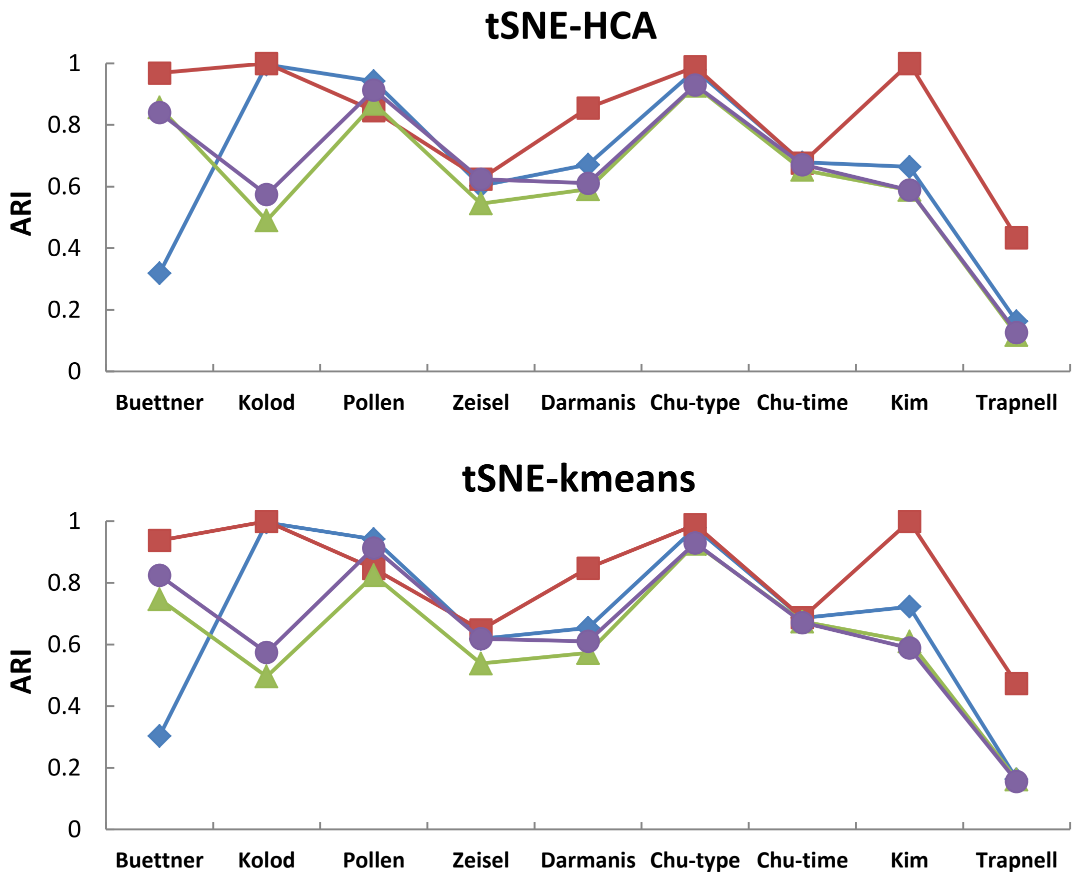

Figure S12. The clustering performance of original GEM, imputed GEM, NDM from original GEM and NDM from imputed GEM on the nine datasets. Hierarchical clustering algorithm (HCA) and k-means were used for comparison. We also performed the clustering to the data that is preprocessed by t-SNE.

Supplementary Note 17: Differential genes in gene expression or network degree between adenocarcinoma and squamous cell carcinoma adjacent normal tissues

We listed the differential genes in gene expression or network degree between adenocarcinoma and squamous cell carcinoma adjacent normal tissues (Wilcoxon rank sum test, FDR < 0.05 and fold change > 2).

Ensembl ID	FDR of gene expression	FDR of network degree	Ensembl ID	FDR of gene expression	FDR of network degree
**ENSG00000090512**	**0.003197**	**0.007644**	ENSG00000213875	**0.015745**	0.708816
ENSG00000107807	0.055148	**0.035364**	ENSG00000214097	0.215530	**0.019641**
ENSG00000108849	0.368530	**0.045265**	ENSG00000214417	**0.034325**	0.806984
ENSG00000118990	**0.043816**	0.097390	ENSG00000216331	0.075561	**0.043294**
ENSG00000122043	0.303478	**0.046362**	ENSG00000217159	0.180773	**0.047935**
**ENSG00000125998**	**0.028002**	**0.028380**	ENSG00000217653	0.365533	**0.025425**
ENSG00000132677	0.703875	**0.019787**	ENSG00000219559	0.118403	**0.026393**
**ENSG00000134757**	**0.000013**	**0.018907**	ENSG00000224299	0.564278	**0.016476**
ENSG00000143556	**0.002407**	0.290305	ENSG00000225513	0.523751	**0.026656**
ENSG00000144785	0.117591	**0.028505**	**ENSG00000226084**	**0.000015**	**0.009083**
**ENSG00000147381**	**0.003110**	**0.005170**	**ENSG00000228697**	**0.032383**	**0.027148**
ENSG00000158874	**0.044069**	0.547680	**ENSG00000229119**	**0.000003**	**0.000919**
ENSG00000159527	0.203220	**0.044516**	ENSG00000231725	0.055468	**0.030999**
ENSG00000163209	**0.000326**	0.086008	ENSG00000232385	**0.001449**	0.551598
ENSG00000163216	**0.004361**	0.843903	ENSG00000233214	**0.046598**	0.468909
ENSG00000169469	**0.000020**	0.092241	ENSG00000233246	0.227560	**0.028999**
ENSG00000169474	**0.000225**	0.258632	ENSG00000233260	0.187237	**0.032592**
**ENSG00000170454**	**0.019020**	**0.024161**	ENSG00000235175	**0.000013**	0.787825
**ENSG00000170465**	**0.004923**	**0.020187**	ENSG00000237409	0.483375	**0.043804**
ENSG00000178363	**0.000016**	0.103138	ENSG00000237490	**0.009014**	0.084218
ENSG00000183242	0.832016	**0.033203**	**ENSG00000237955**	**0.028421**	**0.000968**
**ENSG00000184330**	**0.016564**	**0.015338**	ENSG00000241794	**0.027922**	0.850091
ENSG00000185479	**0.000194**	0.901974	**ENSG00000242113**	**0.005058**	**0.001137**
ENSG00000186832	**0.000255**	0.455530	ENSG00000244712	0.383523	**0.015151**
ENSG00000186847	**0.001649**	0.223682	ENSG00000251377	0.170296	**0.002587**
**ENSG00000187054**	**0.000189**	**0.019431**	ENSG00000255723	0.170214	**0.028800**
**ENSG00000188373**	**0.039188**	**0.009669**	ENSG00000257759	0.136402	**0.019879**
**ENSG00000197641**	**0.015678**	**0.026463**	ENSG00000259303	0.088267	**0.046652**
ENSG00000199676	**0.041426**	0.878395	ENSG00000261587	0.799625	**0.041349**
**ENSG00000203785**	**0.000052**	**0.001433**	ENSG00000263829	0.789961	**0.012973**
ENSG00000205420	**0.000051**	0.270627	ENSG00000265394	0.149375	**0.022218**
ENSG00000205628	0.313880	**0.042657**	ENSG00000273962	**0.018738**	0.085123
ENSG00000213033	0.956406	**0.022251**	**ENSG00000281550**	**0.039444**	**0.040817**
ENSG00000213455	**0.010947**	0.746091	**ENSG00000281591**	**0.032370**	**0.032139**

Supplementary Note 18: The two subtypes divided from squamous cell carcinoma samples

We can divide squamous cell carcinoma samples into two subtypes S1 and S2 based on NDM (Figure 6C in main text), which are listed below.

Subtype S1 (117 samples):

TCGA-18-3409	TCGA-37-3792	TCGA-52-7622	TCGA-60-2716	TCGA-85-6560	TCGA-77-A5GH
TCGA-18-3410	TCGA-37-4129	TCGA-52-7809	TCGA-60-2725	TCGA-85-7950	TCGA-85-A4PA
TCGA-21-1078	TCGA-37-4130	TCGA-52-7812	TCGA-63-6202	TCGA-85-8048	TCGA-85-A50M
TCGA-21-1083	TCGA-37-4132	TCGA-56-6546	TCGA-66-2744	TCGA-85-8288	TCGA-85-A510
TCGA-21-5787	TCGA-37-4135	TCGA-56-7223	TCGA-66-2754	TCGA-85-8352	TCGA-85-A513
TCGA-22-1000	TCGA-37-4141	TCGA-56-7731	TCGA-66-2755	TCGA-85-8584	TCGA-90-A4ED
TCGA-22-1002	TCGA-37-5819	TCGA-56-8083	TCGA-66-2756	TCGA-90-7767	TCGA-90-A4EE
TCGA-22-1005	TCGA-39-5011	TCGA-56-8201	TCGA-66-2757	TCGA-90-7964	TCGA-90-A59Q
TCGA-22-1016	TCGA-39-5034	TCGA-56-8307	TCGA-66-2769	TCGA-94-7943	TCGA-96-A4JL
TCGA-22-1017	TCGA-39-5039	TCGA-56-8309	TCGA-66-2785	TCGA-94-8491	TCGA-98-A53B
TCGA-22-4594	TCGA-39-5040	TCGA-56-8624	TCGA-66-2786	TCGA-96-7545	TCGA-98-A53C
TCGA-22-4596	TCGA-43-2576	TCGA-56-8626	TCGA-66-2789	TCGA-98-7454	TCGA-98-A53D
TCGA-22-5472	TCGA-43-2578	TCGA-56-8628	TCGA-68-8250	TCGA-98-8022	TCGA-98-A53H
TCGA-22-5480	TCGA-43-2581	TCGA-58-8386	TCGA-68-8251	TCGA-98-8023	TCGA-L3-A4E7
TCGA-22-5481	TCGA-43-5668	TCGA-58-8388	TCGA-77-6842	TCGA-33-AASB	TCGA-NK-A5D1
TCGA-22-5483	TCGA-43-7656	TCGA-60-2695	TCGA-77-7335	TCGA-56-A49D	TCGA-O2-A52Q
TCGA-33-4587	TCGA-43-7658	TCGA-60-2697	TCGA-77-7465	TCGA-56-A4ZJ	TCGA-O2-A5IB
TCGA-33-6737	TCGA-46-3766	TCGA-60-2706	TCGA-77-8007	TCGA-63-A5MM	
TCGA-34-2608	TCGA-46-3767	TCGA-60-2714	TCGA-77-8131	TCGA-63-A5MN	
TCGA-34-5234	TCGA-46-3769	TCGA-60-2715	TCGA-85-6175	TCGA-77-A5FZ	

Subtype S2 (384 samples):

TCGA-18-3406	TCGA-33-6738	TCGA-56-7822	TCGA-66-2790	TCGA-85-8479	TCGA-63-A5MV
TCGA-18-3407	TCGA-34-2596	TCGA-56-7823	TCGA-66-2791	TCGA-85-8481	TCGA-63-A5MW
TCGA-18-3408	TCGA-34-2600	TCGA-56-8082	TCGA-66-2792	TCGA-85-8580	TCGA-63-A5MY
TCGA-18-3411	TCGA-34-5231	TCGA-56-8304	TCGA-66-2793	TCGA-85-8582	TCGA-68-A59I
TCGA-18-3412	TCGA-34-5232	TCGA-56-8305	TCGA-66-2794	TCGA-85-8664	TCGA-68-A59J
TCGA-18-3414	TCGA-34-5236	TCGA-56-8308	TCGA-66-2795	TCGA-85-8666	TCGA-6A-AB49
TCGA-18-3415	TCGA-34-5239	TCGA-56-8503	TCGA-66-2800	TCGA-90-6837	TCGA-77-A5G1
TCGA-18-3416	TCGA-34-5240	TCGA-56-8504	TCGA-68-7755	TCGA-90-7766	TCGA-77-A5G3
TCGA-18-3417	TCGA-34-5241	TCGA-56-8622	TCGA-68-7756	TCGA-90-7769	TCGA-77-A5G6
TCGA-18-3419	TCGA-34-5927	TCGA-56-8623	TCGA-68-7757	TCGA-92-7340	TCGA-77-A5G7
TCGA-18-3421	TCGA-34-5928	TCGA-56-8625	TCGA-70-6722	TCGA-92-7341	TCGA-77-A5G8
TCGA-18-4083	TCGA-34-5929	TCGA-56-8629	TCGA-70-6723	TCGA-92-8063	TCGA-77-A5GA
TCGA-18-4086	TCGA-34-7107	TCGA-58-8387	TCGA-77-6843	TCGA-92-8064	TCGA-77-A5GB
TCGA-18-4721	TCGA-34-8454	TCGA-58-8390	TCGA-77-6844	TCGA-92-8065	TCGA-77-A5GF
TCGA-18-5592	TCGA-34-8455	TCGA-58-8391	TCGA-77-6845	TCGA-94-7033	TCGA-85-A4CL

TCGA-18-5595	TCGA-34-8456	TCGA-58-8392	TCGA-77-7138	TCGA-94-7557	TCGA-85-A4CN
TCGA-21-1070	TCGA-37-3783	TCGA-58-8393	TCGA-77-7139	TCGA-94-8035	TCGA-85-A4JB
TCGA-21-1071	TCGA-37-3789	TCGA-60-2696	TCGA-77-7140	TCGA-94-8490	TCGA-85-A4JC
TCGA-21-1072	TCGA-37-4133	TCGA-60-2698	TCGA-77-7141	TCGA-96-7544	TCGA-85-A4QQ
TCGA-21-1075	TCGA-39-5016	TCGA-60-2703	TCGA-77-7142	TCGA-96-8169	TCGA-85-A4QR
TCGA-21-1076	TCGA-39-5019	TCGA-60-2704	TCGA-77-7337	TCGA-96-8170	TCGA-85-A50Z
TCGA-21-1077	TCGA-39-5021	TCGA-60-2707	TCGA-77-7338	TCGA-98-8020	TCGA-85-A511
TCGA-21-1079	TCGA-39-5022	TCGA-60-2708	TCGA-77-7463	TCGA-98-8021	TCGA-85-A512
TCGA-21-1080	TCGA-39-5024	TCGA-60-2709	TCGA-77-8008	TCGA-21-A5DI	TCGA-85-A53L
TCGA-21-1081	TCGA-39-5027	TCGA-60-2710	TCGA-77-8009	TCGA-22-A5C4	TCGA-85-A5B5
TCGA-21-1082	TCGA-39-5028	TCGA-60-2711	TCGA-77-8128	TCGA-33-A4WN	TCGA-94-A4VJ
TCGA-21-5782	TCGA-39-5029	TCGA-60-2712	TCGA-77-8130	TCGA-33-A5GW	TCGA-94-A5I4
TCGA-21-5783	TCGA-39-5030	TCGA-60-2713	TCGA-77-8133	TCGA-33-AAS8	TCGA-94-A5I6
TCGA-21-5784	TCGA-39-5031	TCGA-60-2719	TCGA-77-8136	TCGA-33-AASD	TCGA-96-A4JK
TCGA-21-5786	TCGA-39-5035	TCGA-60-2720	TCGA-77-8138	TCGA-33-AASI	TCGA-98-A538
TCGA-22-0940	TCGA-39-5036	TCGA-60-2721	TCGA-77-8139	TCGA-33-AASJ	TCGA-98-A539
TCGA-22-0944	TCGA-39-5037	TCGA-60-2722	TCGA-77-8140	TCGA-33-AASL	TCGA-98-A53A
TCGA-22-1011	TCGA-43-3394	TCGA-60-2723	TCGA-77-8143	TCGA-34-A5IX	TCGA-98-A53I
TCGA-22-1012	TCGA-43-3920	TCGA-60-2724	TCGA-77-8144	TCGA-37-A5EL	TCGA-98-A53J
TCGA-22-4591	TCGA-43-5670	TCGA-60-2726	TCGA-77-8145	TCGA-37-A5EM	TCGA-J1-A4AH
TCGA-22-4593	TCGA-43-6143	TCGA-63-5128	TCGA-77-8146	TCGA-37-A5EN	TCGA-L3-A524
TCGA-22-4595	TCGA-43-6647	TCGA-63-5131	TCGA-77-8148	TCGA-43-A474	TCGA-LA-A446
TCGA-22-4599	TCGA-43-6770	TCGA-63-7020	TCGA-77-8150	TCGA-43-A475	TCGA-LA-A7SW
TCGA-22-4601	TCGA-43-6771	TCGA-63-7021	TCGA-77-8153	TCGA-43-A56U	TCGA-MF-A522
TCGA-22-4604	TCGA-43-6773	TCGA-63-7022	TCGA-77-8154	TCGA-43-A56V	TCGA-NC-A5HD
TCGA-22-4605	TCGA-43-7657	TCGA-63-7023	TCGA-77-8156	TCGA-56-A4BW	TCGA-NC-A5HE
TCGA-22-4607	TCGA-43-8115	TCGA-66-2727	TCGA-79-5596	TCGA-56-A4BX	TCGA-NC-A5HF
TCGA-22-4609	TCGA-43-8116	TCGA-66-2734	TCGA-85-6561	TCGA-56-A4BY	TCGA-NC-A5HG
TCGA-22-4613	TCGA-43-8118	TCGA-66-2737	TCGA-85-6798	TCGA-56-A4ZK	TCGA-NC-A5HH
TCGA-22-5471	TCGA-46-3765	TCGA-66-2742	TCGA-85-7696	TCGA-56-A5DR	TCGA-NC-A5HI
TCGA-22-5473	TCGA-46-3768	TCGA-66-2753	TCGA-85-7697	TCGA-56-A5DS	TCGA-NC-A5HJ
TCGA-22-5474	TCGA-46-6025	TCGA-66-2758	TCGA-85-7698	TCGA-56-A62T	TCGA-NC-A5HK
TCGA-22-5477	TCGA-46-6026	TCGA-66-2759	TCGA-85-7699	TCGA-58-A46J	TCGA-NC-A5HL
TCGA-22-5478	TCGA-51-4079	TCGA-66-2763	TCGA-85-7710	TCGA-58-A46K	TCGA-NC-A5HM
TCGA-22-5479	TCGA-51-4080	TCGA-66-2765	TCGA-85-7843	TCGA-58-A46L	TCGA-NC-A5HN
TCGA-22-5482	TCGA-51-4081	TCGA-66-2766	TCGA-85-7844	TCGA-58-A46M	TCGA-NC-A5HO
TCGA-22-5485	TCGA-51-6867	TCGA-66-2767	TCGA-85-8049	TCGA-58-A46N	TCGA-NC-A5HP
TCGA-22-5489	TCGA-52-7810	TCGA-66-2768	TCGA-85-8052	TCGA-63-A5M9	TCGA-NC-A5HQ
TCGA-22-5491	TCGA-52-7811	TCGA-66-2770	TCGA-85-8070	TCGA-63-A5MB	TCGA-NC-A5HR
TCGA-22-5492	TCGA-56-1622	TCGA-66-2771	TCGA-85-8071	TCGA-63-A5MG	TCGA-NC-A5HT
TCGA-33-4532	TCGA-56-5897	TCGA-66-2773	TCGA-85-8072	TCGA-63-A5MH	TCGA-NK-A5CR
TCGA-33-4533	TCGA-56-5898	TCGA-66-2777	TCGA-85-8276	TCGA-63-A5MI	TCGA-NK-A5CT
TCGA-33-4538	TCGA-56-6545	TCGA-66-2778	TCGA-85-8277	TCGA-63-A5MJ	TCGA-NK-A5CX
TCGA-33-4547	TCGA-56-7221	TCGA-66-2780	TCGA-85-8287	TCGA-63-A5ML	TCGA-NK-A7XE

TCGA-33-4566	TCGA-56-7222	TCGA-66-2781	TCGA-85-8350	TCGA-63-A5MP	TCGA-O2-A52N
TCGA-33-4582	TCGA-56-7579	TCGA-66-2782	TCGA-85-8351	TCGA-63-A5MR	TCGA-O2-A52S
TCGA-33-4583	TCGA-56-7580	TCGA-66-2783	TCGA-85-8353	TCGA-63-A5MS	TCGA-O2-A52V
TCGA-33-4586	TCGA-56-7582	TCGA-66-2787	TCGA-85-8354	TCGA-63-A5MT	TCGA-O2-A52W
TCGA-33-4589	TCGA-56-7730	TCGA-66-2788	TCGA-85-8355	TCGA-63-A5MU	TCGA-XC-AA0X

Supplementary Note 19: Survival analyses based on "dark" genes

We can find some "dark" genes from TCGA lung cancer bulk RNA-seq data. These "dark" genes have no differential expressions between lung cancer samples and normal samples (FDR of Wilcoxon rank-sum test > 0.05), which are ignored by the traditional methods. But by our CSN method, they have significantly differential network degrees, which are considered important at a network level. As shown in Figure S13, although those "dark" genes have no differential expressions, they can divide the adenocarcinoma samples into two parts based on the normalized network degree. The survival analyses of those "dark" genes indicate the significant difference between the two parts, which implies their prognosis ability, and have potential applications in precision medicine or personalized treatment.

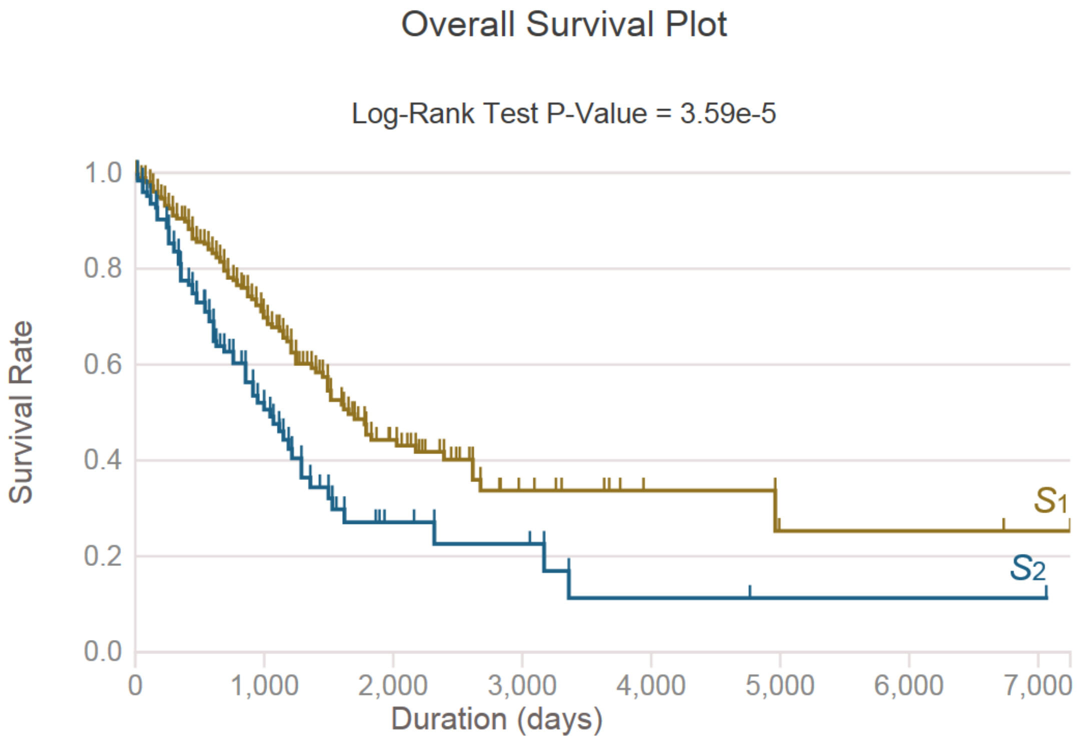

(A) AC007638.2 (novel transcript, antisense to HLF). S_1 (391 samples): normalized degree > 8, S_2 (133 samples): normalized degree ≤ 8

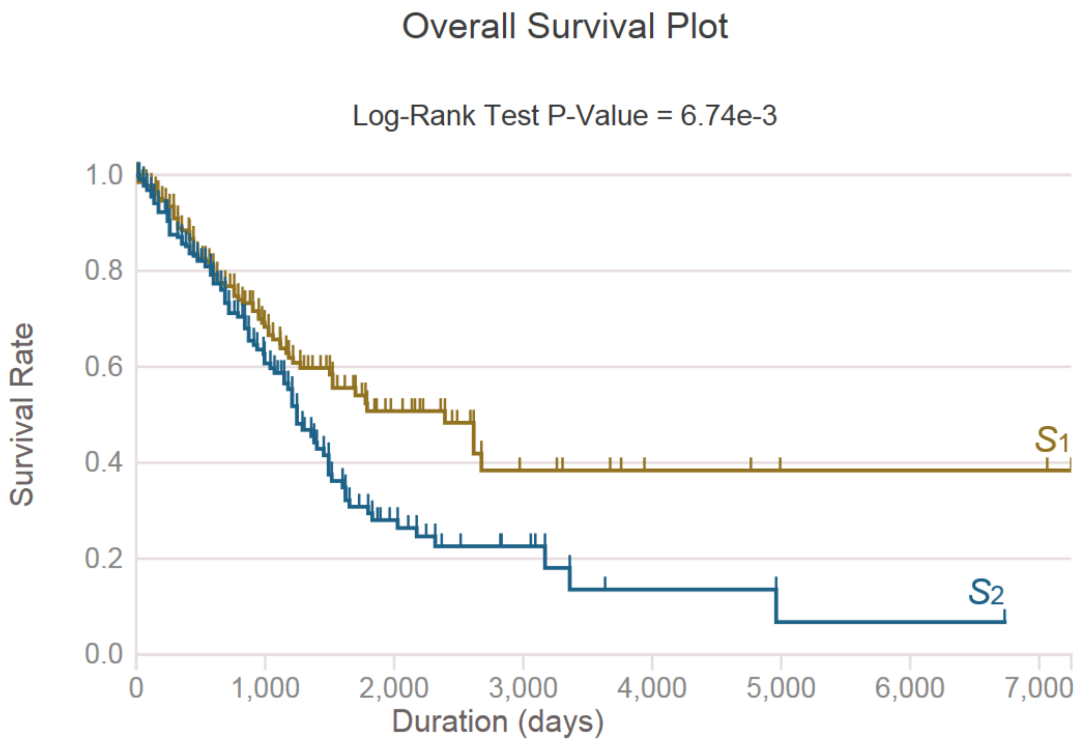

(B) AC092574.2 (novel transcript, antisense to ZNF721). S_1 (288 samples): normalized degree > 14, S_2 (236 samples): normalized degree ≤ 14

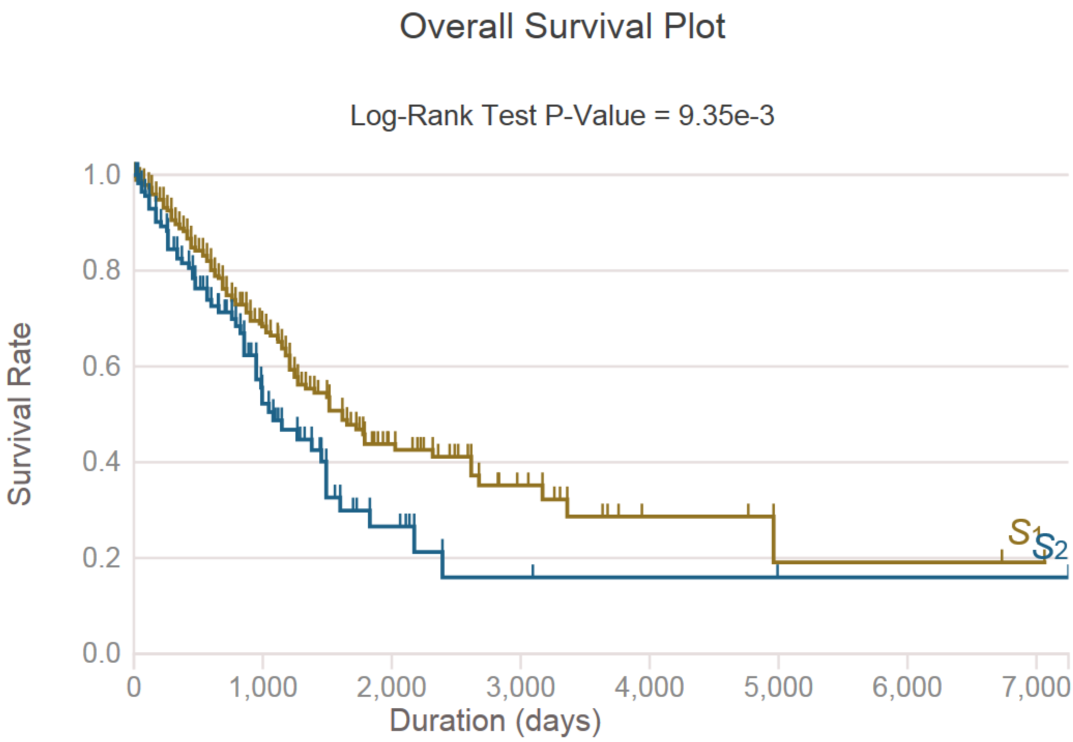

(C) AC002563.1 (novel transcript, antisense to CIT). S_1 (402 samples): normalized degree > 7, S_2 (122 samples): normalized degree ≤ 7

Figure S13. Survival analyses of three "dark" genes.

Supplementary Note 20: Source Code (MATLAB)

(1) Output is CSNs

Construction of cell-specific networks from gene expression matrix

```
function csn = csnet(data,c,alpha,boxsize,weighted)
%Construction of cell-specific networks
%The function performs the transformation from gene expression matrix to
%cell-specific network (csn).
%data: Gene expression matrix, rows = genes, columns = cells
%c: Construct the CSNs for all cells, set c = [] (Default);
%   Construct the CSN for cell k, set  c = k
%alpha: Significant level (eg. 0.001, 0.01, 0.05 ...)
%       larger alpha leads to more edges, Default = 0.01
%boxsize: Size of neighborhood, Default = 0.1
%weighted: 1  edge is weighted
%          0  edge is not weighted (Default)
%csn: Cell-specific network, the kth CSN is in csn{k}
%     rows = genes, columns = genes
%%%%%%%%%%%%%%%%%%%%%%%%%%%%%%%%%%%%%%%%%%%%%%%%%%%%%%
% Too many cells or genes may lead to out of memory. %
%%%%%%%%%%%%%%%%%%%%%%%%%%%%%%%%%%%%%%%%%%%%%%%%%%%%%%

if nargin < 5 || isempty(weighted)
    weighted = 0;
end
if nargin < 4 || isempty(boxsize)
    boxsize = 0.1;
end
if nargin <3 || isempty(alpha)
    alpha = 0.01;
end

[n1,n2] = size(data);
if nargin <2 || isempty(c)
    c = 1 : n2;
end

%Define the neighborhood of each plot
upper = zeros(n1,n2);
lower = zeros(n1,n2);
for i = 1 : n1
    [s1,s2] = sort(data(i,:));
    n3 = n2-sum(sign(s1));
```

```
    h = round(boxsize/2*sum(sign(s1)));
    k = 1;
    while k <= n2
        s = 0;
        while k+s+1 <= n2 && s1(k+s+1) == s1(k)
            s = s+1;
        end
        if s >= h
            upper(i,s2(k:k+s)) = data(i,s2(k));
            lower(i,s2(k:k+s)) = data(i,s2(k));
        else
            upper(i,s2(k:k+s)) = data(i,s2(min(n2,k+s+h)));
            lower(i,s2(k:k+s)) = data(i,s2(max(n3*(n3>h)+1,k-h)));
        end
        k = k+s+1;
    end
end

%Construction of cell-specific network
csn = cell(1,n2);
B = zeros(n1,n2);
p = -icdf('norm',alpha,0,1);
for k = c
    for j = 1 : n2
        B(:,j) = data(:,j) <= upper(:,k) & data(:,j) >= lower(:,k);
    end
    a = sum(B,2);
    d = (B*B'*n2-a*a')./sqrt((a*a').*((n2-a)*(n2-a)')/(n2-1)+eps);
    d(1 : n1+1 : end) = 0;
    if weighted
        csn{k} = d.*(d > 0);
    else
        csn{k} = sparse(d > p);
    end
    disp(['Cell ' num2str(k) ' is completed']);
end
```

(2) Output is NDM

Construction of network degree matrix from gene expression matrix

```
function ndm = csndm(data,alpha,boxsize,normalize)
%Construction of network degree matrix
%The function performs the transformation from gene expression matrix to
```

```
%network degree matrix (ndm).
%data: Gene expression matrix (TPM/FPKM/count), rows = genes, columns =
cells
%alpha: Significant level (eg. 0.001, 0.01, 0.05 ...), Default = 0.01
%boxsize: Size of neighborhood, Default = 0.1 (nx(k) = ny(k) = 0.1*n)
%normalize: 1  result is normalized (Default); 0  result is not normalized

if nargin < 4 || isempty(normalize)
    normalize = 1;
end
if nargin < 3 || isempty(boxsize)
    boxsize = 0.1;
end
if nargin <2 || isempty(alpha)
    alpha = 0.01;
end

%Define the neighborhood of each plot
[n1,n2] = size(data);
upper = zeros(n1,n2);
lower = zeros(n1,n2);
for i = 1 : n1
    [s1,s2] = sort(data(i,:));
    n0 = n2-sum(sign(s1));
    h = round(boxsize/2*sum(sign(s1)));
    k = 1;
    while k <= n2
        s = 0;
        while k+s+1 <= n2 && s1(k+s+1) == s1(k)
            s = s+1;
        end
        if s >= h
            upper(i,s2(k:k+s)) = data(i,s2(k));
            lower(i,s2(k:k+s)) = data(i,s2(k));
        else
            upper(i,s2(k:k+s)) = data(i,s2(min(n2,k+s+h)));
            lower(i,s2(k:k+s)) = data(i,s2(max(n0*(n0>h)+1,k-h)));
        end
        k = k+s+1;
    end
end

%If gene expression matrix is sparse, use the sparse matrix will accelerate
%the calculation and reduce memory footprint
```

```
%data = sparse(data); upper = sparse(upper); lower = sparse(lower);

%Construction of network degree matrix
ndm = zeros(n1,n2);
B = zeros(n1,n2);
p = -icdf('norm',alpha,0,1);
for k = 1 : n2
    for j = 1 : n2
        B(:,j) = data(:,j) <= upper(:,k) & data(:,j) >= lower(:,k) &
data(:,k);
    end
    %B = sparse(B);
    a = sum(B,2);
    csn = (B*B'*n2-a*a')./sqrt((a*a').*((n2-a)*(n2-a)')/(n2-1)+eps);
    csn = (csn > p);
    ndm(:,k) = sum(csn,2) - diag(csn);
    disp(['Cell ' num2str(k) ' is completed']);
end

%Normalization of network degree matrix
if normalize
    ndm = bsxfun(@rdivide,ndm,sum(ndm))*mean(sum(sign(ndm)))^2/2000;
end
```

(3) Output is Edge

Normalized statistic of edge *x*-*y* from the expression values of genes *x* and *y*

```
function edge = csnedge(gx,gy,boxsize)
%The normalized statistic of edge x-y
%gx gy: Gene expression values of gene x and gene y
%       If there are n cells, gx and gy are 1-by-n vectors
%boxsize: Size of neighborhood, Default = 0.1
%edge: 1-by-n vector, the normalized statistic of edge x-y in all cells

if nargin < 3
    boxsize = 0.1;
end

%Define the neighborhood of each plot
n = length(gx);
upper = zeros(1,n);
lower = zeros(1,n);
a = zeros(2,n);
```

```
B = cell(1,2);
for i = 1 : 2
    g = gx*(i==1)+gy*(i==2);
    [s1,s2] = sort(g);
    n0 = n-sum(sign(s1));
    h = round(boxsize/2*sum(sign(s1)));
    k = 1;
    while k <= n
        s = 0;
        while k+s+1 <= n && s1(k+s+1) == s1(k)
            s = s+1;
        end
        if s >= h
            upper(s2(k:k+s)) = g(s2(k));
            lower(s2(k:k+s)) = g(s2(k));
        else
            upper(s2(k:k+s)) = g(s2(min(n,k+s+h)));
            lower(s2(k:k+s)) = g(s2(max(n0*(n0>h)+1,k-h)));
        end
        k = k+s+1;
    end

    B{i} = bsxfun(@le,g',upper) & bsxfun(@ge,g',lower);
    a(i,:) = sum(B{i});
end

%Calculate the normalized statistic of edge x-y in all cells
edge = (sum(B{1} & B{2})*n-a(1,:).*a(2,:))./sqrt(a(1,:).*a(2,:) ...
    .*(n-a(1,:)).*(n-a(2,:))/(n-1));
```

References

1. Arthur David and Vassilvitskii, S. (2007) k-means++: the advantages of careful seeding. *SODA '07: Proceedings of the Eighteenth Annual ACM-SIAM Symposium on Discrete Algorithms.*, **11**, 1027-1035.
2. Kaufman, L. and Rousseeuw, P.J. (2009) *Finding Groups in Data: An Introduction to Cluster Analysis*. John Wiley & Sons, Inc., Hoboken, New Jersey.
3. Xu, C. and Su, Z. (2015) Identification of cell types from single-cell transcriptomes using a novel clustering method. *Bioinformatics*, **31**, 1974-1980.
4. Wang, B., Zhu, J., Pierson, E., Ramazzotti, D. and Batzoglou, S. (2017) Visualization and analysis of single-cell RNA-seq data by kernel-based similarity learning. *Nature Methods*, **14**, 414-416.
5. Maaten, L.v.d. and Hinton, G. (2008) Visualizing Data using t-SNE. *Journal of Machine Learning Research*, **9**, 2579-2605.
6. Bendall, S.C., Davis, K.L., Amir, E.-a.D., Tadmor, M.D., Simonds, E.F., Chen, T.J., Shenfeld, D.K., Nolan, G.P. and Pe'er, D. (2014) Single-cell trajectory detection uncovers progression and regulatory coordination in human B cell development. *Cell*, **157**, 714-725.
7. Kim, K.T., Lee, H.W., Lee, H.O., Song, H.J., Jeong, D.E., Shin, S., Kim, H., Shin, Y., Nam, D.H., Jeong, B.C. *et al.* (2016) Application of single-cell RNA sequencing in optimizing a combinatorial therapeutic strategy in metastatic renal cell carcinoma. *Genome Biol.*, **17**.
8. Zeisel, A., Muñoz-Manchado, A.B., Codeluppi, S., Lönnerberg, P., Manno, G.L., Juréus, A., Marques, S., Munguba, H., He, L., Betsholtz, C. *et al.* (2015) Brain structure. Cell types in the mouse cortex and hippocampus revealed by single-cell RNA-seq. *Science*, **347**, 1138-1142.
9. Buettner, F., Natarajan, K.N., Casale, F.P., Proserpio, V., Scialdone, A., Theis, F.J., Teichmann, S.A., Marioni, J.C. and Stegle, O. (2015) Computational analysis of cell-to-cell heterogeneity in single-cell RNA-sequencing data reveals hidden subpopulations of cells. *Nature Biotechnology*, **33**, 155-160.
10. Trapnell, C., Cacchiarelli, D., Grimsby, J., Pokharel, P., Li, S., Morse, M., Lennon, N.J., Livak, K.J., Mikkelsen, T.S. and Rinn, J.L. (2014) The dynamics and regulators of cell fate decisions are revealed by pseudotemporal ordering of single cells. *Nature Biotechnology*, **32**, 381-386.
11. Pollen, A.A., Nowakowski, T.J., Shuga, J., Wang, X., Leyrat, A.A., Lui, J.H., Li, N., Szpankowski, L., Fowler, B., Chen, P. *et al.* (2014) Low-coverage single-cell mRNA sequencing reveals cellular heterogeneity and activated signaling pathways in developing cerebral cortex. *Nature Biotechnology*, **32**, 1053-1058.
12. Xin, Y., Kim, J., Okamoto, H., Ni, M., Wei, Y., Adler, C., Murphy, A.J., Yancopoulos, G.D., Lin, C. and Gromada, J. (2016) RNA Sequencing of Single Human Islet Cells Reveals Type 2 Diabetes Genes. *Cell Metab.*, **24**, 608-615.
13. Kolodziejczyk, A.A., Kim, J.K., Tsang, J.C.H., Ilicic, T., Henriksson, J., Natarajan, K.N., Tuck, A.C., Gao, X., Bühler, M., Liu, P. *et al.* (2015) Single Cell RNA-Sequencing of Pluripotent States Unlocks Modular Transcriptional Variation. *Cell Stem Cell*, **17**, 471-485.
14. Chu, L.-F., Leng, N., Zhang, J., Hou, Z., Mamott, D., Vereide, D.T., Choi, J., Kendziorski, C., Stewart, R. and Thomson, J.A. (2016) Single-cell RNA-seq reveals novel regulators of human embryonic stem cell differentiation to definitive endoderm. *Genome Biology*, **17**, 173.
15. Darmanis, S., Sloan, S.A., Zhang, Y., Enge, M., Caneda, C., Shuer, L.M., Gephart, M.G.H.,

Barres, B.A. and Quake, S.R. (2015) A survey of human brain transcriptome diversity at the single cell level. *Proceedings of the National Academy of Sciences of the United States of America*, **112**, 7285-7290.

16. Shen, L., Qin, K., Wang, D., Zhang, Y., Bai, N., Yang, S., Luo, Y., Xiang, R. and Tan, X. (2014) Overexpression of Oct4 suppresses the metastatic potential of breast cancer cells via Rnd1 downregulation. *Biochim Biophys Acta.*, **1842**, 2087-2095.
17. Covello, K.L., Kehler, J., Yu, H., Gordan, J.D., Arsham, A.M., Hu, C.-J., Labosky, P.A., Simon, M.C. and Keith, B. (2006) HIF-2alpha regulates Oct-4: effects of hypoxia on stem cell function, embryonic development, and tumor growth. *Genes Dev.*, **20**, 557-570.
18. Pan, G., Li, J., Zhou, Y., Zheng, H. and D, D.P. (2006) A negative feedback loop of transcription factors that controls stem cell pluripotency and self-renewal. *FASEB J.*, **20**, 1730-1732.
